# Common genetic risk variants identified in the SPARK cohort support DDHD2 as a candidate risk gene for autism

**DOI:** 10.1038/s41398-020-00953-9

**Published:** 2020-08-03

**Authors:** Nana Matoba, Dan Liang, Huaigu Sun, Nil Aygün, Jessica C. McAfee, Jessica E. Davis, Laura M. Raffield, Huijun Qian, Joseph Piven, Yun Li, Sriam Kosuri, Hyejung Won, Jason L. Stein

**Affiliations:** 1 Department of Genetics, University of North Carolina at Chapel Hill, Chapel Hill, NC 27599 USA; 2 UNC Neuroscience Center, University of North Carolina at Chapel Hill, Chapel Hill, NC 27599 USA; 3 Department of Chemistry and Biochemistry, University of California, Los Angeles, Los Angeles, CA 90095 USA; 4 UCLA-DOE Institute for Genomics and Proteomics, University of California, Los Angeles, Los Angeles, CA 90095 USA; 5 Molecular Biology Institute, University of California, Los Angeles, Los Angeles, CA 90095 USA; 6 Quantitative and Computational Biology Institute, University of California, Los Angeles, Los Angeles, CA 90095 USA; 7 Eli and Edythe Broad Center of Regenerative Medicine and Stem Cell Research, University of California, Los Angeles, Los Angeles, CA 90095 USA; 8 Jonsson Comprehensive Cancer Center, University of California, Los Angeles, Los Angeles, CA 90095 USA; 9 Department of Statistics and Operations Research, University of North Carolina at Chapel Hill, Chapel Hill, NC 27599 USA; 10 Department of Psychiatry and the Carolina Institute for Developmental Disabilities, University of North Carolina at Chapel Hill, Chapel Hill, NC 27599 USA; 11 Department of Biostatistics, University of North Carolina at Chapel Hill, Chapel Hill, NC 27599 USA; 12 Department of Computer Science, University of North Carolina at Chapel Hill, Chapel Hill, NC 27599 USA

**Keywords:** Genetics, Autism spectrum disorders

## Abstract

Autism spectrum disorder (ASD) is a highly heritable neurodevelopmental disorder. Large genetically informative cohorts of individuals with ASD have led to the identification of a limited number of common genome-wide significant (GWS) risk loci to date. However, many more common genetic variants are expected to contribute to ASD risk given the high heritability. Here, we performed a genome-wide association study (GWAS) on 6222 case-pseudocontrol pairs from the Simons Foundation Powering Autism Research for Knowledge (SPARK) dataset to identify additional common genetic risk factors and molecular mechanisms underlying risk for ASD. We identified one novel GWS locus from the SPARK GWAS and four significant loci, including an additional novel locus from meta-analysis with a previous GWAS. We replicated the previous observation of significant enrichment of ASD heritability within regulatory regions of the developing cortex, indicating that disruption of gene regulation during neurodevelopment is critical for ASD risk. We further employed a massively parallel reporter assay (MPRA) and identified a putative causal variant at the novel locus from SPARK GWAS with strong impacts on gene regulation (rs7001340). Expression quantitative trait loci data demonstrated an association between the risk allele and decreased expression of *DDHD2* (DDHD domain containing 2) in both adult and prenatal brains. In conclusion, by integrating genetic association data with multi-omic gene regulatory annotations and experimental validation, we fine-mapped a causal risk variant and demonstrated that *DDHD2* is a novel gene associated with ASD risk.

## Introduction

Autism spectrum disorder (ASD) is a common neurodevelopmental disorder characterized by characteristic social deficits, as well as ritualistic behaviors^1^. Because ASD is highly heritable (~50–80%)^2–6^, a number of studies have been conducted to identify both rare and common genetic variants contributing to risk for ASD. While previous studies have successfully identified rare de novo and rare inherited presumed loss of function mutations leading to risk for ASD^7–14^, these de novo variants do not explain the large heritability and therefore are missing an important component of ASD risk.

To identify common inherited genetic risk factors, genome-wide association studies (GWAS) have accumulated over 18,000 individuals with ASD and have begun discovering genome-wide significant (GWS) loci that explain some of the inherited risks for ASD^15^. The previously discovered three GWS ASD susceptibility loci from the discovery sample of the iPSYCH-PGC study together explain only 0.13% of the liability for autism risk, whereas all common variants are estimated to explain 11.8% of liability^15^. Therefore, there are more common risk variants to be discovered, which requires larger sample sizes to provide sufficient power to detect risk variants of small effect^16–18^. The newly established genetic cohort, SPARK (Simons Foundation Powering Autism Research for Knowledge) (https://sparkforautism.org/), is planning to collect and analyze data from 50,000 individuals with ASD^19^. SPARK has recently released genotype data for over 8000 families or singletons with ASD, which we utilize here to increase the power of ASD GWAS.

Once we identify GWS loci, the critical next step is to understand their biological impact. This is especially challenging because most GWAS identified loci for neurodevelopmental disorders and other traits are located in poorly annotated non-coding regions with presumed gene regulatory function^20^. In addition, most loci are comprised of multiple single nucleotide polymorphisms (SNPs) that are often inherited together, which makes it difficult to identify the true causal variant(s) and their regulatory effects^21,22^. To overcome these problems, various experimental validation tools have been developed^23–25^. One of these tools, a massively parallel reporter assay (MPRA), simultaneously evaluates allelic effects on enhancer activity for many variants. In this assay, exogenous DNA constructs, harboring risk and protective alleles at an associated variant, drive the expression of a barcoded transcript. Differences in barcode counts between the risk and protective alleles indicate the regulatory function of that variant^24,25^. This assay thus demonstrates the regulatory potential of individual SNPs and provides evidence of causal variants within an associated locus.

Though fine-mapping approaches can suggest causal variants at a locus, they cannot identify target genes affected by those variants. Several approaches are designed to link variants to genes they regulate including expression quantitative trait loci (eQTL)^26–28^, as well as chromatin interaction (via Hi–C) assays^29–31^. Recently, we developed Hi–C coupled MAGMA (H-MAGMA) which predicts genes associated with the target phenotype by integrating long-range chromatin interaction with GWAS summary statistics^32^. Together with existing eQTL resources in the adult and fetal cortex^33,34^, it is possible to link variants associated with risk for ASD to target genes and functional pathways.

In this study, we increase the sample size of existing ASD GWAS by adding 6222 cases-pseudocontrol pairs from the genetically diverse SPARK project. Our analysis identified five loci associated with risk for ASD, including two novel loci. For one novel locus identified, we used an MPRA to identify the causal variant within the locus. Further, we integrated multi-level functional genomic data obtained from the developing brain, including eQTLs, chromatin interactions, and regulatory elements, to identify *DDHD2* as a candidate gene involved in ASD etiology at the MPRA-validated locus.

## Methods and materials

This study (analysis of this publicly available dataset) was reviewed by the Office of Human Research Ethics at UNC, which has determined that this study does not constitute human subjects research as defined under federal regulations [45 CFR 46.102 (d or f) and 21 CFR 56.102(c)(e)(l)] and does not require IRB approval.

### SPARK dataset

SPARK participants who received any of the following diagnoses: autism spectrum disorder [ASD], Asperger syndrome, autism/autistic disorder and pervasive developmental disorder-not otherwise specified (PDD-NOS) were recruited. The samples were enriched for affected individuals whose parents were also available to participate. Participants registered for SPARK online at www.SPARKforAutism.org or at 25 clinical sites across the country by completing questionnaires on medical history and social communication as described here: https://www.sfari.org/spark-phenotypic-measures/. Thus, case status is based on patient/parent-report.

In this study, participants were drawn from the SPARK 27K release (20190501 ver.) through SFARIBase (https://www.sfari.org/resource/sfari-base/), which included 27,290 individuals (who were genotyped with a SNP array and/or whole-exome sequencing [WES]) with phenotype information such as sex, diagnosis, and cognitive impairment. The data included probands and their family members if applicable (e.g., 3192 quads (2798 families with unaffected siblings, 394 with multiple affected siblings), 2486 trios, and 2448 duos) (Supplementary Fig. 1). Individuals overlapping with either Autism Sequencing Consortium (ASC) cohorts or the Simons Simplex Collection (SSC) were excluded by SPARK. Twenty families in this release overlapped with the Simon’s Variations in Individuals Project (SVIP) cohort and were subsequently removed for the genome-wide association analysis (Supplementary Fig. 2) since the SVIP cohort has targeted probands with 16p11.2 deletions. We also obtained whole-exome sequencing (WES) data to estimate the imputation accuracy. Details on genotyping and whole-exome sequencing data, and pre-imputation quality control are provided in Supplementary Methods.

### Genotype phasing and imputation

Phasing was performed using EAGLE v2.4.1^35^ (https://data.broadinstitute.org/alkesgroup/Eagle/) within SPARK samples. Before making pseudocontrols, we removed two individuals, one each from two pairs of monozygotic twins with Identity-By-Descent (PI_HAT) > 0.9, by selecting the individual with lower call rates. We then defined pseudocontrols by PLINK 1.9^36^ (www.cog-genomics.org/plink/1.9/) for trios by selecting the alleles not inherited from the parents to the case^37^. We re-phased all SPARK samples that passed our QC measures with pseudocontrols. Imputation was performed on the Michigan imputation server^38^ (https://imputationserver.sph.umich.edu/index.html). Since SPARK participants are genetically diverse, we imputed genotypes using the Trans-Omics for Precision Medicine (TOPMed) Freeze 5b (https://www.nhlbiwgs.org/) reference panel which consists of 125,568 haplotypes from multiple ancestries^39,40^. Imputation accuracy relative to WES was assessed using a similar approach to previous work^41^ (Supplementary Fig. 3) as described in Supplementary Methods.

### Genome-wide association analysis and meta-analysis with iPSYCH-PGC study

We tested association within the SPARK all case-pseudocontrol pairs (full dataset; Supplementary Table 1) using PLINK2 generalized linear model (--glm) for SNPs with MAF ≥ 0.01 and imputation quality score from minimac4 (R2) > 0.5 (Supplementary Fig. 3). In this model, we did not include any covariates since cases and pseudocontrols are matched on environmental variables and genetic ancestry. We performed secondary GWAS analyses by subsetting to only specific ancestry groups. We called ancestry using multidimensional scaling (MDS) analysis with 988 HapMap3 individuals and one random case from each trio (Supplementary Fig. 4, Supplementary Table 2). Ancestry of individuals from SPARK was called as European, African or East Asian ancestries if they were within 5 standard deviations of defined HapMap3 population (CEU/TSI; YRI/LWK; or CHB/CHD/JPT, respectively) centroids in MDS dimensions 1 and 2. Population-specific GWASs were carried out using the same association model as described above for the SPARK all ancestries dataset. Meta-analyses with iPSYCH-PGC study^15^ were performed by METAL (release 2018–08–28)^42^. Additional information for iPSYCH-PGC summary statistics is provided in Supplementary Methods.

### Investigation of pleiotropic effects for ASD loci

The pleiotropic effects of identified loci were investigated for phenotypes available in the NHGRI/EBI GWAS catalog (downloaded October 22, 2019)^43^ (Supplementary Methods).

### Linkage disequilibrium score regression analysis

LD SCore regression (LDSC) (v1.0.0)^44,45^ was used to estimate genome-wide SNP based heritability, heritability enrichment of tissue/cell-type specific epigenetic states, and genetic correlation across phenotypes for GWAS meta-analysis results (Supplementary Methods). Prior to the analyses, we filtered SNPs to those found in HapMap3 and converted to LDSC input files (.sumstats.gz) using munge_sumstats.py. The pre-computed LD scores for Europeans were obtained from https://data.broadinstitute.org/alkesgroup/LDSCORE/eur_w_ld_chr.tar.bz2. For all LDSC analyses, we used individuals from European ancestry as described in the “Genome-wide association analysis (GWAS)” section above.

### Estimating polygenic risk score

Polygenic risk scores (PRSs) were calculated based on the iPSYCH-PGC study^15^ using PRSice-2^46^ (https://www.prsice.info/). Details on generation of PRS, sex-stratified and family-type PRS, and parental origin PRS analyses are provided in Supplementary Methods.

### H-MAGMA

SNP to Ensembl gene annotation was carried out by Hi–C coupled MAGMA (H-MAGMA) (https://github.com/thewonlab/H-MAGMA) by leveraging chromatin-interaction generated from fetal and adult brain Hi–C^33,47^ as previously described^32^. Details on H-MAGMA and functional analyses of H-MAGMA genes are provided in Supplementary Methods.

### Construction of a massively parallel reporter assay (MPRA) library

Because the novel SPARK associated locus (chr8:38.19M–chr8:38.45M) was also detected in a previous schizophrenia GWAS which is better powered, we obtained a set of credible SNPs for the locus based on schizophrenia GWAS results^48^ (see Supplementary Methods). Ninety-eight credible SNPs were detected in this locus. We obtained 150 bp sequences that flank each credible SNP with the SNP at the center (74 bp + 75 bp). Because each SNP has risk and protective alleles, this resulted in 196 total alleles to be tested. We seeded HEK293 cells (ATCC® CRL-11268™) in 6 wells (total 6 replicates) to be 70–90% confluent at transfection. We used lipofectamine 2000 (Invitrogen cat#11668) with our final MPRA library following the manufacturer’s instructions. Additional information for construction of MPRA library is available in Supplementary Methods. MPRA data was analyzed by the mpra package in R^49,50^ (https://github.com/hansenlab/mpra) with more details in Supplementary Methods.

### Functional annotation of rs7001340 locus with multi-omic datasets

To investigate the target gene(s) affected by allelic variation at rs7001340, we used two expression quantitative trait loci (eQTL) datasets derived from fetal cortical brain tissue^34^ and adult dorsolateral prefrontal cortex^33^. We also used chromatin accessibility profiles from primary human neural progenitor cells and their differentiated neuronal progeny^51^, as well as HEK293T cells (GSM1008573)^52^. Further information is provided in Supplementary Methods.

## Results

### GWAS in SPARK dataset identified a new locus associated with ASD risk

**Fig. 1:**
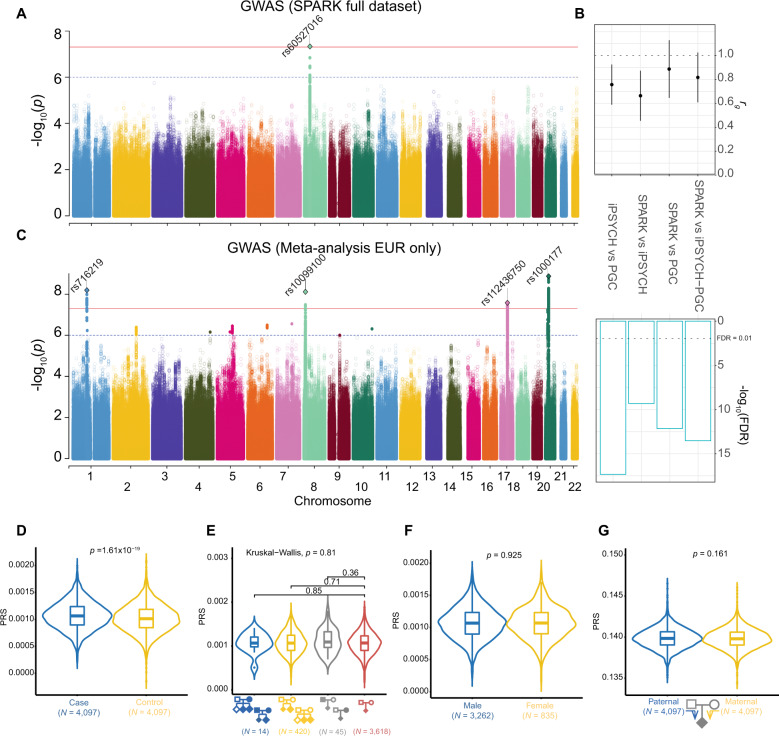
Genome-wide association of ASD in the SPARK dataset. **a** GWAS result from SPARK full dataset (*N*_case+pseudocontrol_ = 12,444). **b** Genetic correlations across ASD GWAS. From left to right, iPSYCH versus PGC^53^, SPARK EUR versus iPSYCH, SPARK EUR versus PGC and SPARK EUR versus iPSYCH-PGC study^15^. **c** GWAS results from the meta-analysis (SPARK European population and iPSYCH-PGC, *N*_max_case+control_ = 55,420). For Manhattan plots (**a**, **c**), the *x*-axes indicate the chromosomal position and y-axes indicate the significance of associations. The blue and red lines denote the significance threshold at suggestive (*P* < 1 × 10^–6^) and significant (*P* < 5 × 10^–8^) levels. SNPs with *P* < 1 × 10^–6^ are shown as a filled circle. Rs number indicates index SNPs from independent loci (1 MB apart from each other) at *P* < 1 × 10^–8^). Index SNPs at *P* < 5 × 10^–8^ are shown as diamonds. **d** Common variant risk burden is higher in cases compared to pseudocontrols. **e** Comparison of PRS across family types (from left to right, families with multiple affected children with affected parent(s), multiple affected children with unaffected parents, one affected child with affected parent(s), and one affected child with unaffected parents) shows no evidence for a higher common variant burden in multiplex families. **f** Comparison of PRS between male and female probands shows no evidence of enrichment of common variants impacting risk for ASD in females. **g** There is no evidence for a difference in the transmission of common variant risk burden from mother versus father.

We obtained genotype and clinical diagnosis of ASD via self-report or parent-report from 27,290 individuals who participated in the SPARK project^19^. The majority of data comprised families, including those where both parents and multiple children were genotyped (quads; *N* = 3192 families), where both parents and one child were genotyped (trios; *N* = 2486 families), or where one parent and one child were genotyped (duos; *N* = 2448 families) (Supplementary Fig. 1). Only 68 individuals were ascertained without family members (singletons). After genotyping quality control (Supplementary Methods), 375,918 variants from 26,883 individuals were retained. Because the SPARK dataset did not genotype unrelated controls, we created pseudocontrols from the alleles not transmitted from parents to probands^37^. Case-pseudocontrol design requires genotyping of both parents, so singletons and duos were excluded from the analysis. Due to the diverse ancestry in the cohort (Supplementary Fig. 4, Supplementary Table 1), genotypes of all individuals including pseudocontrols were imputed to a diverse reference panel (TOPMed Freeze 5b reference panel consisting of 125,568 haplotypes). After imputation quality control (Methods; Supplementary Fig. 2, 3), 8,992,756 autosomal SNPs were tested for association in 6222 case-pseudocontrol pairs (SPARK full dataset) consisting of 4956 males and 1266 females from multiple ancestries including European (*N* = 4535), African (*N* = 37), East Asian (*N* = 83) and other ancestries/admixed individuals (*N* = 1567) (Supplementary Fig. 2, Supplementary Table 2). We observed no inflation of test statistics (λ_GC_ = 1.00) (Supplementary Fig. 5), indicating population stratification was well-controlled when using this case-pseudocontrol design. We identified two SNPs at one locus (index SNP: rs60527016-C; OR = 0.84, *P* = 4.70 × 10^–8^) at genome-wide significance (*P* < 5.0 × 10^–8^) (Fig. 1a, Table 1, Supplementary Table 3), which were supported by the previous largest ASD GWAS^15^ (OR = 0.95, *P* = 0.0047) derived from the PGC and iPSYCH cohorts (Supplementary Fig. 6).

**Table 1:** Genome-wide significant loci associated with ASD risk.

SNP^a^	Position(hg38)	EA	OA	EAF	SPARK		iPSYCH + PGC		Meta(EUR)^b^	
					OR(95%CI)	*P*	OR(95%CI)	*P*	OR(95%CI)	*P*
Genome-wide significant loci (*P* < 5 × 10^–8^)										
rs716219	1:96104001	T	C	0.34	1.08 (1.03–1.14)	0.003	1.08 (1.05–1.11)	3.99 × 10^–7^	1.08 (1.05–1.11)	**6.42×10**^**–9**^
rs10099100	8:10719265	C	G	0.31	1.08 (1.02–1.14)	0.008	1.09 (1.06–1.12)	**1.07×10**^**–8**^	1.08 (1.05–1.11)	**7.65×10**^**–9**^
rs60527016	8:38442106	C	T	0.21	0.84 (0.79–0.90)	**4.70×10**^**−8**^	0.95 (0.92−0.99)	0.00466	0.93 (0.91–0.96)	3.05 × 10^−6^
rs112436750	17:45887763	A	AT	0.21	1.07 (1.01−1.14)	0.027	1.09 (1.05−1.12)	1.23 × 10^−6^	1.09 (1.06–1.12)	**2.62×10**^**−8**^
rs1000177	20:21252560	T	C	0.24	1.08 (1.02−1.15)	0.014	1.10 (1.07−1.14)	**3.32×10**^**−9**^	1.09 (1.06–1.13)	**1.34×10**^**−9**^
Suggestive loci (5 × 10^−8^ ≤ *P* < 1 × 10^−6^)										
rs6701243	1:98627228	A	C	0.38	0.99 (0.94−1.00)	0.610	0.93 (0.90−0.96)	3.07 × 10^−7^	0.94 (0.91–0.96)	5.90 × 10^--7^
rs6743102	2:158521946	G	A	0.34	0.94 (0.89−0.99)	0.021	0.94 (0.91–0.97)	8.99 × 10^−6^	0.94 (0.91–0.96)	4.07 × 10^−7^
rs33966416	4:170285452	CA	C	0.50	0.95 (0.90−1.00)	0.038	0.94 (0.91–0.96)	2.73 × 10^−6^	0.94 (0.92−0.96)	6.99 × 10^−7^
rs4916723	5:88558577	A	C	0.40	1.10 (1.00–1.10)	0.062	1.07 (1.04–1.10)	1.92 × 10^−6^	1.07 (1.04–1.09)	6.90 × 10^−7^
rs416223	5:104655775	C	A	0.40	1.00 (0.96–1.10)	0.730	1.07 (1.04–1.10)	3.84 × 10^−7^	1.07 (1.04–1.09)	3.56 × 10^−7^
rs67248478	6:134711094	C	T	0.34	0.94 (0.90–1.10)	0.032	0.94 (0.91–0.96)	3.22 × 10^−6^	0.94 (0.91–0.96)	3.22 × 10^−7^
rs76569799	9:73565191	C	T	0.15	1.10 (0.99–1.10)	0.076	1.09 (1.05–1.13)	3.90 × 10^−6^	1.08 (1.05–1.12)	9.99 × 10^−7^
rs4750990	10:128689762	T	C	0.36	1.00 (0.98–1.10)	0.250	1.07 (1.04–1.10)	1.37 × 10^−6^	1.07 (1.04–1.09)	4.89 × 10^−7^
rs2224274	20:14780101	C	T	0.43	1.00 (0.97–1.10)	0.310	1.07 (1.04–1.10)	2.86 × 10^−7^	1.07 (1.05–1.10)	5.56 × 10^−8^

Genome-wide significant and suggestive loci in any of the GWAS analyses and meta-analysis of SPARK European ancestries and iPSYCH+PGC participants are shown.

*EA* effect allele, *OA* other allele, *EAF* effect allele frequency in SPARK full dataset.

^a^Index SNPs from loci that survived genome-wide significance in any of the GWASs including meta-analysis.

^b^Meta-analysis of SPARK European ancestries and iPSYCH+PGC.

*P*-values < 5 × 10^−8^ are shown in bold.

### Replication of genetic risk factors for ASD

Given the phenotypic heterogeneity of ASD and potential technical differences such as genotyping platforms or data processing, we assessed the replication of genetic risk factors across cohorts by comparing previous major ASD studies including PGC and iPSYCH cohort^15,53^ with the SPARK dataset subset to individuals of European descent (EUR) (Fig. 1b). Although each study included multiple ASD subtypes including ASD from DSM5, Asperger’s, autism/autistic disorder, and Pervasive Developmental Disorder–Not otherwise specified (PDD-NOS) from DSM IV, and approaches differed across these samples from requiring community diagnosis to best-estimate diagnosis based on standardized assessment, we obtained high genetic correlations between the SPARK EUR dataset and the largest iPSYCH-PGC GWAS (*r*_g_ = 0.82; *P* = 5.27 × 10^–14^), suggesting the genetic risk factors for autism are largely shared among different ASD GWAS and are generalizable despite differences in diagnostic criteria and batch effects.

We next performed meta-analysis with SPARK EUR samples and iPSYCH-PGC samples (*N*_case_ = 18,381 and *N*_control_ = 27,969) to maximize power. The meta-analysis identified four additional loci associated with risk for ASD (Supplementary Figs. 7–10). These included three previously reported loci^15^ and one novel locus on chromosome 17, where a gene-based test from the iPSYCH-PGC study has previously shown association with risk for ASD^15^ (Fig. 1c, Table 1, Supplementary Fig. 9). This novel locus was also reported to be associated with more than 60 phenotypes including neuroticism^54–58^, educational attainment^59^ and intracranial volume^60^ (index SNPs *r*^*2*^ > 0.8 in 1 KG EUR) (Supplementary Table 4), indicating highly pleiotropic effects. The SNP based heritability in SPARK EUR samples was estimated (*h*^*2*^_G_) to be 0.117 (s.e. = 0.0082) for population prevalence of 0.012^15,61^ which was comparable with the previous report (*h*^*2*^_G_ = 0.118; s.e. = 0.010)^15^.

The generalization of effects across ancestries for the five index SNPs identified (Table 1) was examined (Supplementary Fig. 6, and Supplementary Table 5). The association results from the cross-ancestry dataset were mainly driven by the European population, as expected given the larger sampling from this population. We found that some regions showed differences in allele frequencies based on population. For example, rs10099100 was more common in European and African populations (MAF = 0.33, 0.39 from tested samples, respectively) than in East Asians (MAF = 0.02 from tested samples), necessitating a further investigation of genetic risk factors for ASD in populations of diverse ancestry^62,63^.

The generalization of genetic effects on risk for ASD was also confirmed by polygenic risk scores (PRSs) derived from the iPSYCH-PGC GWAS that showed higher scores in SPARK EUR cases (*N* = 4097) compared to pseudocontrols (*N* = 4097) (*P* = 1.61 × 10^–19^; *p* value threshold = 0.01; Nagelkerke’s *R*^*2*^ = 1.4%) (Fig. 1d, Supplementary Fig. 11).

### Investigation of common variant burden impacting risk for ASD

We next used PRSs to compare common variant risk burden among family types, sex, and parent of origin (Fig. 1e-g). Because ASD families with multiple affected siblings were shown to have different segregation patterns compared with simplex families that have a higher burden of de novo mutations^11,64,65^, we compared the distribution of PRSs across four family types (Fig. 1e, Supplementary Table 1). Our results showed no evidence for a difference in common variant burden impacting risk for ASD in multiplex families as compared to simplex families. We note that multiplex/simplex status was indicated by either enrollment or self-report in a questionnaire and may be underestimated due to missing survey data.

As the prevalence of ASD is higher in males than in females (OR = 4.20)^66^, and previous studies have reported that females with ASD have a higher burden of de novo variants^9,67–69^, we also investigated the potential contribution of common variants to the female protective effect by comparing PRS between sexes. We did not find evidence that ASD common variant risk burden differs between females and males (Fig. 1f).

A previous study hypothesized that a new mutation in a mother, who is less susceptible to developing autism because of the female protective effect, may be more likely to transmit risk factors to their children with ASD^70^. We, therefore, examined the over-transmission of common variant risk for ASD from mother to offspring. We found no evidence of the over-transmission of common variant risk burden from either mothers or fathers to their affected children (Fig. 1g).

### Contribution of cortical development to risk for ASD

Previous studies suggest an important role of brain development in ASD^15,71^. To characterize tissue types relevant to risk for ASD, we next evaluated heritability enrichment within active enhancer or promoter regions in different tissues^72^ (Supplementary Fig. 12A, Table 5). Significant enrichment of heritability was observed in regulatory elements of brain germinal matrix, as well as primary cultured neurospheres from the fetal cortex (FDR = 0.004 and 0.015, respectively, Supplementary Table 6), suggesting that disruption of gene regulation in these tissues increases the risk for ASD. We further examined SNP heritability in the developing cortex using differentially accessible peaks between the neuron-enriched cortical plate and the progenitor-enriched germinal zone^73^ (Supplementary Fig. 12B). We found significant enrichment in peaks more accessible in the germinal zone (FDR = 0.008), but not in the cortical plate, replicating previous reports that genetically mediated alterations in cortical development play a crucial role in ASD etiology^15^.

### H-MAGMA identified genes and pathways impacting risk for ASD

**Fig. 2:**
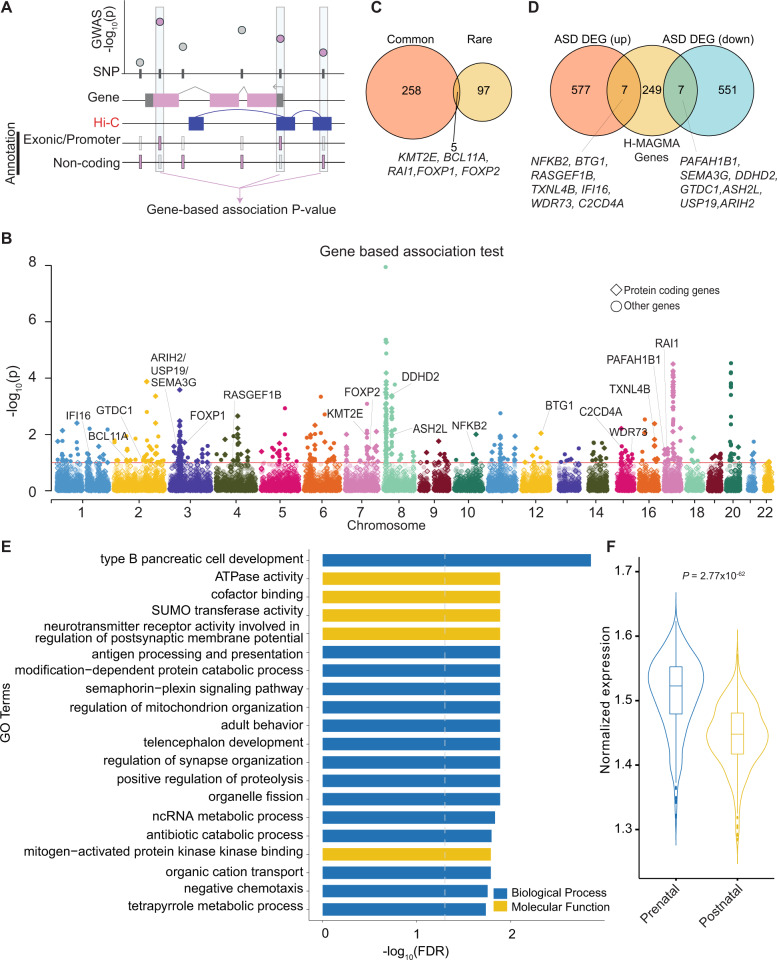
H-MAGMA identified 263 protein-coding genes linked to ASD. **a** Schematic diagram of H-MAGMA. SNP based association *P*-values were aggregated to gene-based *P*-values using positional information, as well as chromatin interaction in the fetal brain. **b** Gene-based association results from H-MAGMA. The *x*-axis indicates the start position of genes (hg19). **c** Overlap of ASD risk genes harboring common and rare variants^74^. **d** Overlapped genes with differential expression from post-mortem brains in individuals with ASD patients and neurotypical controls^77^. **e** Gene ontologies enriched for ASD linked genes (top 20). **f** Developmental expression pattern of ASD risk genes^118^.

To identify genes associated with risk for ASD from meta-analysis (EUR only), we applied Hi–C coupled MAGMA (H-MAGMA)^32^, which aggregates SNP-level *P*-values into a gene-level association statistic with an additional assignment of non-coding SNPs to their chromatin-interacting target genes generated from fetal brain Hi–C^47^ (Fig. 2a). We identified 567 genes associated with ASD (FDR < 0.1), including 263 protein coding genes (Fig. 2b, Supplementary Table 7). Five genes implicated from common variant evidence (*KMT2E*, *RAI1*, *BCL11A, FOXP1*, and *FOXP2*) also harbored an excess of rare variants associated with ASD^74^. This overlap between rare and common ASD risk variants was more than expected by chance (hypergeometric *P* = 0.01; Fig. 2c), corroborating previous findings that common and rare variation converge on the same genes and pathways^32,75,76^. We also found that 14 H-MAGMA genes were also differentially expressed in the post-mortem cortex between individuals with ASD and neurotypical controls (upregulated in ASD: *NFKB2*, *BTG1*, *RASGEF1B*, *TXNL4B*, *IFI16*, *WDR73*, and *C2CD4A*; downregulated in ASD: *PAFAH1B1*, *SEMA3G*, *DDHD2*, *GTDC1 ASH2L, USP19*, and *ARIH2*; FDR < 0.05)^77^ (Fig. 2d). Rank-based gene ontology enrichment analysis^78^ suggested that ASD risk genes were enriched in 188 terms including telencephalon development and regulation of synapse organization (Fig. 2E, Supplementary Tables 8, 9).

**Fig. 3:**
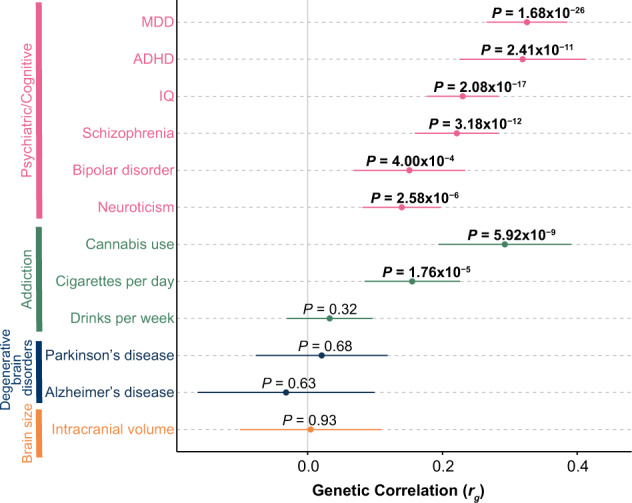
Genetic correlation of ASD against twelve brain and behavioral phenotypes. The *x*-axis represents an estimate of the genetic correlation (*r*_g_). Error bars represent the 95% confidence interval. *P*-values at FDR < 0.05 are shown in bold. MDD major depressive disorder, ADHD attention-deficit/hyperactivity disorder.

Since heritability enrichment analyses suggested genetically mediated impacts on cortical development contribute to ASD risk (Supplementary Fig. 12), we explored whether the expression level of ASD risk genes from H-MAGMA is different between prenatal and postnatal cortex. In this analysis, we combined H-MAGMA genes from either adult or fetal brain Hi–C (Supplementary Fig. 13) to ensure that the enrichment is not driven by the use of Hi–C from only one developmental time period, such as observing higher prenatal expression levels of H-MAGMA identified ASD risk genes exclusively due to the use of fetal brain Hi–C (Supplementary Table 7,10). As shown previously^15,32^, we found ASD risk genes exhibited higher expression in the prenatal cortex as compared to the postnatal cortex (*P* = 2.77 × 10^–62^) (Fig. 3f). In particular, the expression level of ASD risk genes was highest between 20 and 30 post-conception weeks (Supplementary Fig. 14, Supplementary Table 11). Taken together, our results demonstrate common risk variants for ASD play an important role in the developing cortex.

### Genetic correlation between ASD and 12 brain and behavioral phenotypes

Both epidemiological studies and genetic studies suggested the phenotypic comorbidity^79–82^ or genetic correlation^15,83^ of ASD with various brain and behavioral phenotypes. Thus, we evaluated the pleiotropic effect of ASD risk SNPs with twelve other brain and behavioral phenotypes^48,57,60,84–91^ (Fig. 3, Supplementary Table 12). We observed a novel genetic correlation between ASD and cigarettes per day (*r*_g_ = 0.16, *P* = 8.80 × 10^–5^), indicating a partially shared genetic basis between risk for ASD and addictive smoking behavior. We also replicated positive genetic correlations previously detected for seven phenotypes (FDR < 0.05)^15^, providing further support for a shared genetic basis of multiple neuropsychiatric disorders^83,92^.

### Functional validation to fine-map causal variants and prioritize genes

**Fig. 4:**
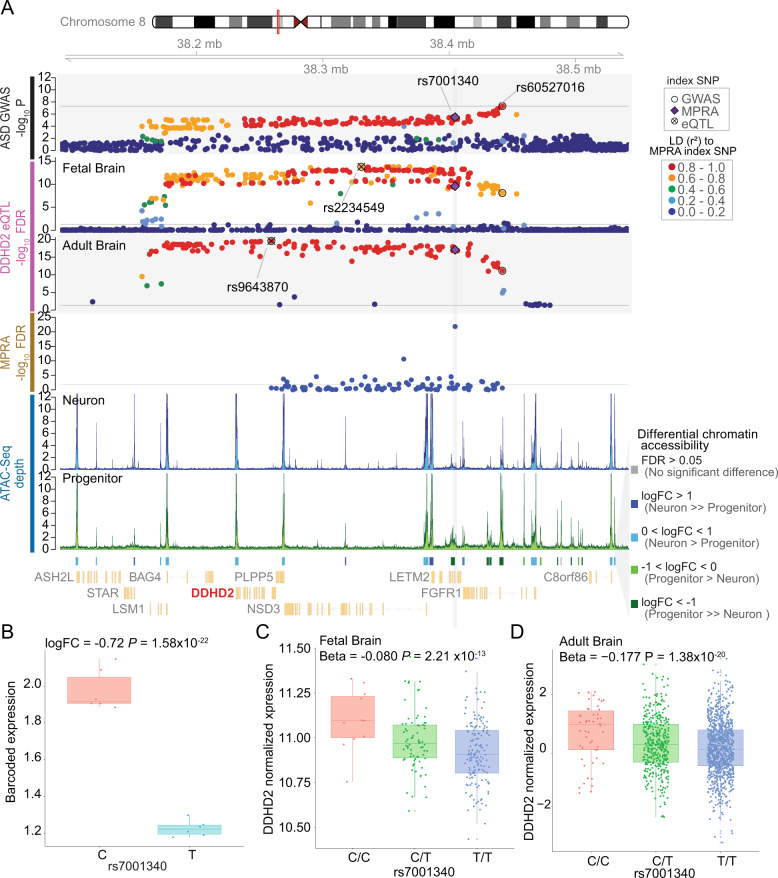
Identification of putative causal variant and gene impacting risk for ASD. **a** Annotated locus plot near rs60527016 ASD risk index variant, from top panel to bottom, ASD associations within SPARK full dataset (*n* = 6222 case-pseudocontrol pairs), eQTL for *DDHD2* in fetal brains (*n* = 235) and adult brain (*n* = 1387), MPRA expression (*n* = 6), ATAC-seq averaged depth in neuron (*n* = 61) and progenitor (*n* = 73). Differential open chromatin accessibility peaks from ATAC-seq, and gene model (NCBI Refseq). LD was calculated to rs7001340 within SPARK parents of cases, fetal brain donors, or 1 KG EUR and colored accordingly. **b** The barcoded expression level of mRNA based from each allele at rs7001340 from the MPRA experiment. **c** The expression level of DDHD2 by rs7001340 genotypes in the fetal brain. **d** The expression level of DDHD2 by rs7001340 genotypes in adult brain. Individuals with allele dosage (0–0.1 as C/C, 0.9–1.1 as C/T, 1.9–2.0 as T/T) are shown. For **b** to **d**, ASD risk allele for rs7001340 is T and protective allele is C.

Interestingly, the novel locus identified by the SPARK full dataset (rs60527016 at chr8:38.19M–chr8:38.45M, Figs. 1a, 4a) was previously identified as a pleiotropic locus in a recent cross-disorder meta-analysis on eight psychiatric disorders^83^, as well as a schizophrenia GWAS^48^ (Supplementary Fig. 15). This locus was not only associated with ASD but also with schizophrenia, bipolar disorder and obsessive-compulsive disorder (OCD), suggesting that understanding the regulatory mechanism at this locus may reveal the basis for pleiotropic effects across psychiatric disorders. The index SNP (rs60527016) was located within a 300 kb LD block (*r*^*2*^ > 0.5 in SPARK full dataset) that contains seven genes (Fig. 4a). To prioritize causal variants within this locus, we performed a massively parallel reporter assay (MPRA)^24,25^ on 98 credible SNPs in this region in HEK 293 cells (Supplementary Fig. 15, 16, 17). MPRA measures barcoded transcriptional activity driven by each allele in a high-throughput fashion (Supplementary Fig. 16). Surprisingly, SNP rs7001340 exhibited the strongest allelic difference in barcoded expression (*P* = 1.51 × 10^–24^) even though it is 37 kb away from the GWAS index SNP (*r*^*2*^ = 0.85 with the index SNP in SPARK full dataset) (Fig. 4a, b, Supplementary Table 13), demonstrating the regulatory potential of this SNP and suggesting its causal role in psychiatric disorders, including ASD. While MPRA was performed in HEK cells, the SNP was located in a regulatory element present in both HEK cells and neural progenitors, with higher chromatin accessibility in human neural progenitors compared with postmitotic neurons^51^ (Fig. 4a, Supplementary Fig. 17), indicating its regulatory potential in the developing brain. The risk allele (T) at this SNP was associated with downregulation of barcoded expression in MPRA (Fig. 4b, Supplementary Fig. 16), and was predicted to disrupt two transcription factor binding motifs^93^ (TBX1 and SMARCC1) (Supplementary Fig. 18), providing a possible mechanism of action of this variant. We next investigated potential target genes impacted by regulatory changes at this SNP by using eQTL data from fetal^34^ and adult brain tissues^33^. Expression levels of three eGenes were significantly associated with this SNP (*DDHD2* from the fetal brain and *DDHD2*, *LSM1*, *LETM2* from the adult brain) (Fig. 4a, Supplementary Fig. 19). Of these three genes, two genes (*DDHD2*, *LETM2*) showed the direction of the effect expected from the MPRA result (risk allele downregulates the eGene). It is of note that *DDHD2* was identified in both fetal and adult brain eQTL datasets (beta = −0.080, *P* = 2.212 × 10^–13^; beta = −0.177, *P* = 1.38 × 10^–20^, respectively; Fig. 4c, d). We further validated the association between *DDHD2* and ASD by additional transcriptome wide association study (TWAS) in the brain (PrediXcan^94–96^ and FUSION^97^) (Supplementary Fig. 20)^28,98–101^. Notably, *DDHD2* was also significantly downregulated in the post-mortem cortex of individuals with autism (logFC = −0.28, FDR = 0.013), providing an added layer of evidence supporting its role in ASD risk^77^. *DDHD2* was also identified by H-MAGMA (Fig. 2f), and a copy number variation (CNV) containing *DDHD2* (deletions) was found in proband-sibling pairs with discordant social-behavior phenotypes^102^. Collectively, by integrating existing multi-level functional genomic resources and an experimental fine-mapping approach using MPRA, we suggest *DDHD2* as a strong candidate gene impacting risk for ASD.

## Discussion

In this study, we increased sample sizes for ASD GWAS to *N*_case(max) _= 24,063, *N*_control(max) _= 34,191 and identified five loci associated with risk for ASD (four from European only meta-analysis, one locus from SPARK project alone), including two new loci (marked by index SNPs rs60527016 and rs112436750). These loci have pleiotropic effects on multiple psychiatric disorders including schizophrenia (for rs60527016 and rs112436750), bipolar disorder, and OCD (for rs60527016).

Using a PRS derived from a previous study^15^, we found enrichment of risk variants in SPARK cases, indicating the contribution of common genetic risk factors to ASD is consistent across cohorts. However, despite several hypotheses that rare variants associated with risk for ASD are enriched in certain subgroups of individuals with ASD, such as in females compared to males (female protective effect)^9,67–69,103^, multiplex families compared to simplex families^11,64,65^, or maternal alleles compared to paternal alleles^10,70^, we do not find evidence to support the increased burden of common risk variants within those subgroups. This result indicates potential rare and common variant differences in contribution to subgroup risk for ASD. However, it is notable that similar to GWAS in other neuropsychiatric disorders^104,105^, PRS explained only a small percent of variance in risk (1.4%). Moreover, given the small sample size of specific subgroups (*N* = 835 in female whereas *N* = 3262 in male, *N* = 14 for families with multiple affected children versus *N* = 3618 with one affected children), our study may have limited power to identify the differences among subgroups. Thus, a larger sample size would be warranted to compare the difference in the role of common variants within these categories.

Identifying locations in the genome associated with risk for ASD does not in itself lead to insights into what tissues or developmental time points are crucial for the etiology of ASD. Here, by integrating SNP association statistics with existing annotations of regulatory elements active during specific developmental time periods or within specific brain regions, we found an excess of common genetic risk for ASD in the fetal brain regulatory elements (brain germinal matrix and primary cultured neurospheres from the fetal cortex), and progenitor enriched germinal zone of the developing cortex, confirming previous findings that alterations of gene regulation in the prenatal cortex play a key role in ASD etiology^15^.

To further understand genes leading to risk for ASD, we applied a recently developed platform, H-MAGMA^32^ and identified 263 putative candidate protein-coding risk genes. H-MAGMA genes are highly expressed in the prenatal brain, similar to the enrichment of ASD risk genes with rare variations during neurodevelopment^106^. This result suggests potential molecular convergence regardless of classes of mutation, which is supported by five genes (previously identified *KMT2E* and newly identified *RAI1*, *BCL11A*, *FOXP1*, and *FOXP2*) that are affected by both rare and common variation.

Since identification of a GWS locus does not elucidate the causal variant(s), we performed MPRA and identified a potential causal SNP (rs7001340) at a novel ASD locus discovered in the SPARK sample. Interestingly, the individual variant with the strongest regulatory effect (rs7001340; *r*^*2*^ = 0.85 with the index SNP in SPARK full dataset) was different from the SNP with the strongest association with ASD (rs60527016), highlighting the importance of experimental validation in identifying causal variants. It should be noted that the regulatory effects of these variants were assessed in non-neural (HEK) cells. Although this regulatory element was found in both HEK cells and neural progenitors (Supplementary Fig. 17), further validation of these effects in ASD-relevant cell types would provide increased confidence in declaring this SNP as causal. The experimentally validated regulatory SNP (rs7001340) is in the intron of *LETM2*, and is also an eQTL for *LETM2*, *LSM1* (247 kb away) and *DDHD2* (173 kb away), indicating that the SNP functions as a distal regulatory element. The risk allele (T) was associated with decreased expression of barcoded transcripts in the MPRA and downregulation of *DDHD2* from eQTL in both fetal and adult brains, implying a consistent direction of the allelic effects on gene regulation. The risk allele showed the same direction of effect for *LETM2* in adult brain tissue, but was not significantly associated in fetal brain tissue (*P*-value = 0.33). Intriguingly, *DDHD2* was also downregulated in the cortex from individuals with ASD compared to neurotypical controls^77^, providing an additional level of support for this gene as a risk factor for ASD. *DDHD2* (DDHD domain-containing protein 2), also known as KIAA0725p, encodes a phospholipase and is localized in the Golgi^107^. *DDHD2* plays a role in the efficient regulation of membrane trafficking between the Golgi and cytosol^107^ and is highly expressed in the brain^108–110^. Mutations in *DDHD2* have been previously found in individuals with spastic paraplegia type 54 (SPG)^110–112^. *Ddhd2* null mice exhibited motor and cognitive impairments^113^, which are frequent comorbidities of ASD^114^. We, therefore, conclude *DDHD2* is a strong candidate risk gene for ASD through multiple lines of evidence.

There is still a large amount of common variant heritability not explained in this study indicating that further increases in sample size will be necessary to explain the common inherited component of ASD risk. While the combination of TOPMed imputation and the case-pseudocontrol study model enabled us to include individuals from multiple ancestries, the case-pseudocontrol model is lower powered compared to case-unscreened control models because a pseudocontrol might have greater liability for ASD than the average individual in the population^115^. In addition, the case-pseudocontrol model cannot incorporate duos or singletons due to the lack of parental genotype information, which resulted in over 2000 individuals with ASD with genotyping information in the SPARK project not being included in our analysis. Moreover, this model has the disadvantage that X-chromosome cannot be analyzed due to lack of untransmitted genotype information from the father. Future studies could potentially solve this problem and also increase power by including all cases available in SPARK and using unscreened population matched controls^116^. Secondly, although we performed population stratified GWAS, the limited number of individuals for some populations (e.g., 37 from AFR and 83 from EAS) may lead to a large standard error in the estimate of the effect size. Also, subsequent analyses including PRS, LDSC regression, and H-MAGMA were limited to individuals from European ancestries only, because most resources and software are designed to be used only within one population, generally European ancestry^117^. Including other ancestries for these analyses will be able to uncover risk factors shared or specific to existing human populations.

In summary, ASD GWAS in the SPARK dataset and meta-analysis with previous GWAS identified two new susceptibility loci. By integrating existing multi-level functional genomic resources and experimental tools such as MPRA and eQTL, we highlight *DDHD2* as a novel high confidence ASD risk gene impacted by a distal common variant within a regulatory element present in neural progenitors of the developing cortex. This strategy can be broadly applied to common variant risk loci of multiple neuropsychiatric disorders to identify causal variant(s), regulatory regions, cell-types, and genes whose misregulation leads to risk for neuropsychiatric disorders.

## Supplementary information

Supplementary Information

Supplementary Table s3

Supplementary Table s4

Supplementary Table s6

Supplementary Table s7

Supplementary Table s8

Supplementary Table s9

Supplementary Table s10

Supplementary Table s11

Supplementary Table s13

## Data Availability

Summary statistics are available at https://bitbucket.org/steinlabunc/spark_asd_sumstats.

## Code availability

Code is available at https://github.com/thewonlab/GWAS_ASD_SPARK.
