# Supplementary material for: Common genetic risk variants identified in the SPARK cohort support DDHD2 as a candidate risk gene for autism
Source: Transl Psychiatry. 2020 Aug 3;10:265. doi: 10.1038/s41398-020-00953-9 (PMC7400671; doi:10.1038/s41398-020-00953-9)
Supplement: Supplementary file 1 — Supplementary Information [file 41398_2020_953_MOESM1_ESM.pdf]

Supplementary Information

Supplementary Figures **2**

Supplementary Figure 1| Examples of family structure in SPARK cohort 2
Supplementary Figure 2| QC flow chart 3
Supplementary Figure 3| Imputation quality assessment 3
Supplementary Figure 4| MDS plots 5
Supplementary Figure 5| QQ plots for SPARK studies 6
Supplementary Figure 6| Forest plots for the index SNPs identified in the SPARK full dataset and iPSYCH+PGC meta-analysis 7
Supplementary Figure 7| Regional association plot for the index SNP (rs716219) on Chromosome 1 from the meta-analysis (EUR) 9
Supplementary Figure 8| Regional association plot for the index SNP (rs10099100) on Chromosome 8 from the meta-analysis (EUR) 10
Supplementary Figure 9| Regional association plot for the index SNP (rs112436750) on Chromosome 17 from the meta-analysis (EUR) 11
Supplementary Figure 10| Regional association plot for the index SNP (rs1000177) on Chromosome 20 from the meta-analysis (EUR) 12
Supplementary Figure 11| Variance explained (Nagelkerke R2) by the ASD PRS 13
Supplementary Figure 12| Partitioned heritability enrichment of tissues implicates cortical development in ASD risk 14
Supplementary Figure 13| Profiling tissue type specific H-MAGMA gene 15
Supplementary Figure 14| Developmental trajectory of ASD risk genes 16
Supplementary Figure 15| Comparison of credible SNPs from Schizophrenia (SZ) and ASD 17
Supplementary Figure 16| Quality check for MPRA experiment 18
Supplementary Figure 17| Conserved chromatin structure in neural progenitors and HEK cells 19
Supplementary Figure 18| Disruption of transcription factor binding motifs by rs7001340 20
Supplementary Figure 19| Expression level of eGenes regulated by rs7001340 21
Supplementary Figure 20| Alternative approaches to test DDHD2 association in multiple tissue types 22

Supplementary Tables **23**

Supplementary Table 1| Study characteristics 23
Supplementary Table 2| HapMap population used for MDS 24
Supplementary Table 3| Assessment of GWAS findings from SPARK dataset related to Table 1. 24
Supplementary Table 4| ASD risk loci have pleiotropic effects on various phenotypes 24
Supplementary Table 5| Association results across populations 24
Supplementary Table 6| Heritability Enrichment in active enhancers or promoters 25
Supplementary Table 7| H-MAGMA Gene list (fetal brain) 25
Supplementary Table 8| Gene ontologies enriched in ASD genes (fetal brain) 26
Supplementary Table 9| Gene ontologies enriched in ASD genes (adult brain) 27
Supplementary Table 10| H-MAGMA Gene list (adult brain) 27
Supplementary Table 11| H-MAGMA Gene WGCNA Module 27

Supplementary Table 12| Genetic correlations between ASD and twelve brain and behavioral phenotypes 28
Supplementary Table 13| MPRA results 28

Supplementary Methods **29**
Genotyping and whole-exome sequencing 29
Pre-imputation quality control for genotype chip data 29
Genotype phasing and imputation 30
Assessment of imputation accuracy 30
Meta-analysis with iPSYCH-PGC study 31
Investigation of pleiotropic effects for ASD loci 31
Estimating polygenic Risk Score 32
Heritability Enrichment Analysis 33
Genetic correlation analysis 33
H-MAGMA 34
Gene ontology enrichment analysis for H-MAGMA ASD genes 34
Overlap with genes derived from H-MAGMA and other studies 35
Developmental expression profiles of ASD linked genes 35
Construction of a Massively Parallel Reporter Assay (MPRA) Library 36
MPRA 38
MPRA analysis 40
Functional annotation of rs7001340 locus with multi-omic datasets 40

References **42**

Supplementary Figures

Supplementary Figure 1| Examples of family structure in SPARK cohort

Examples of family tree showing (**A**) multiple children were genotyped (quads; *N*=3,192 families), (**B**) both parents and one child were genotyped (trios; *N*=2,486 families), and (**C**) one parent and one child were genotyped (duos; *N*=2,448 families). Parent(s) could be either affected or unaffected.

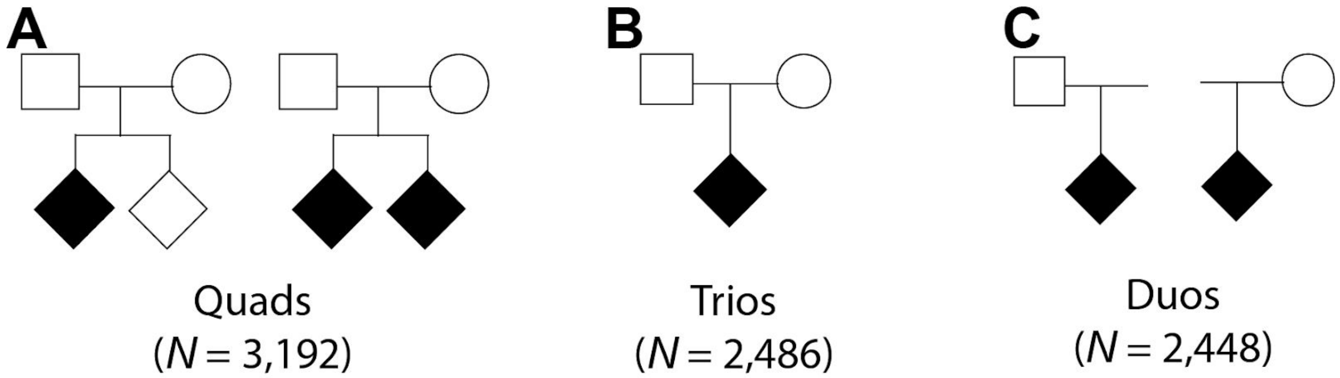

Supplementary Figure 2| QC flow chart

Numbers of variants and samples filtered during the QC step are shown on the left and right sides, respectively. After all QC, we utilized 6,222 trios in the SPARK full dataset.

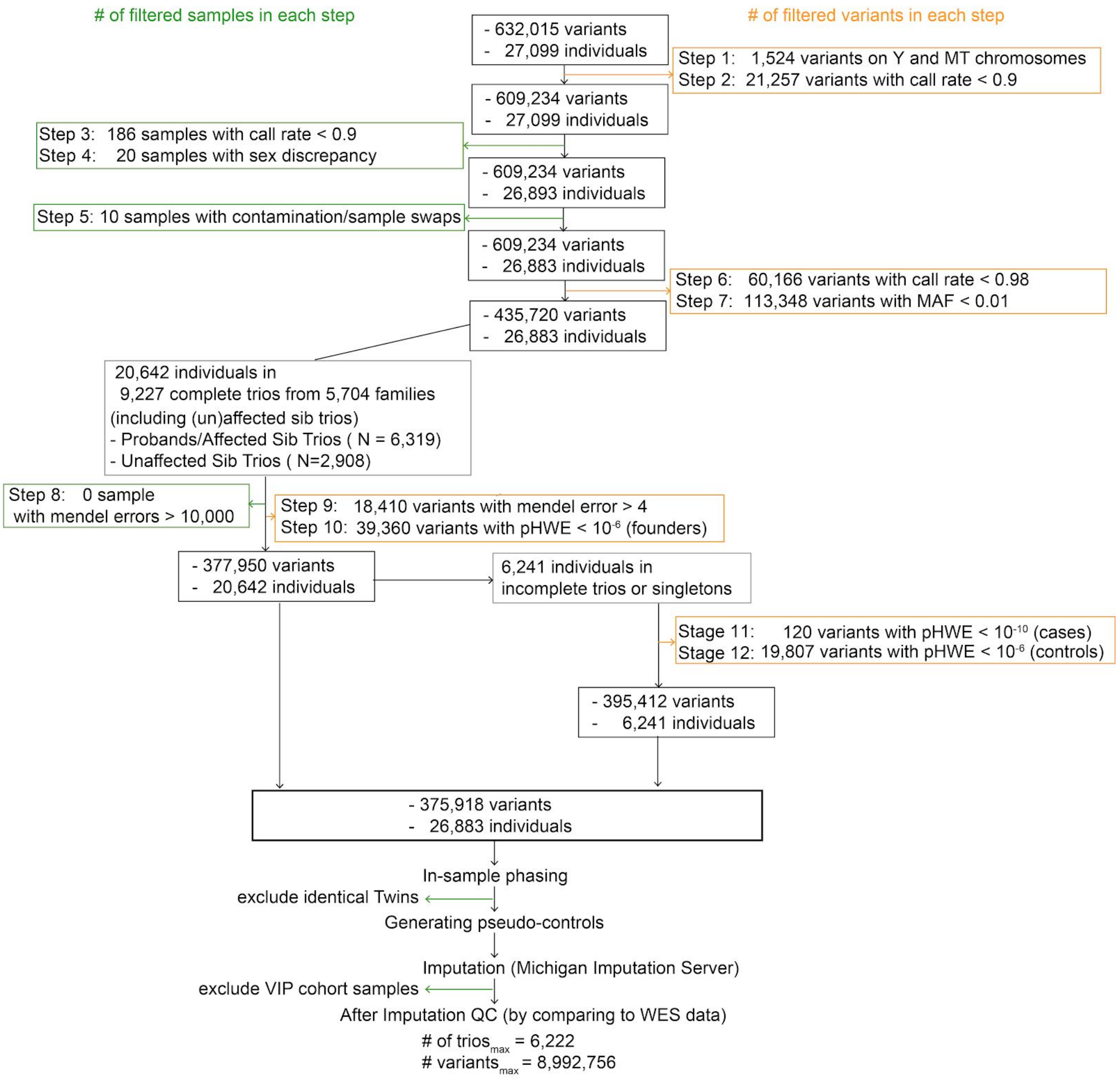

Supplementary Figure 3| Imputation quality assessment

Imputation accuracy was assessed for all individuals by comparing them with whole-exome sequencing data. (**A**) Minimac4 imputed R^2 (y-axis) across minor allele frequency (MAF) bin (x-axis) on chromosome 20. (**B**) Mismatch rate (%) (y-axis) relative to WES binned by minimac4 imputed R^2 (x-axis). (**C**) Distribution of mismatch rate across participants calculated by comparing to WES data. The vertical line indicates mismatch rate at 3% which was used as an exclusion criteria (72 individuals were removed).

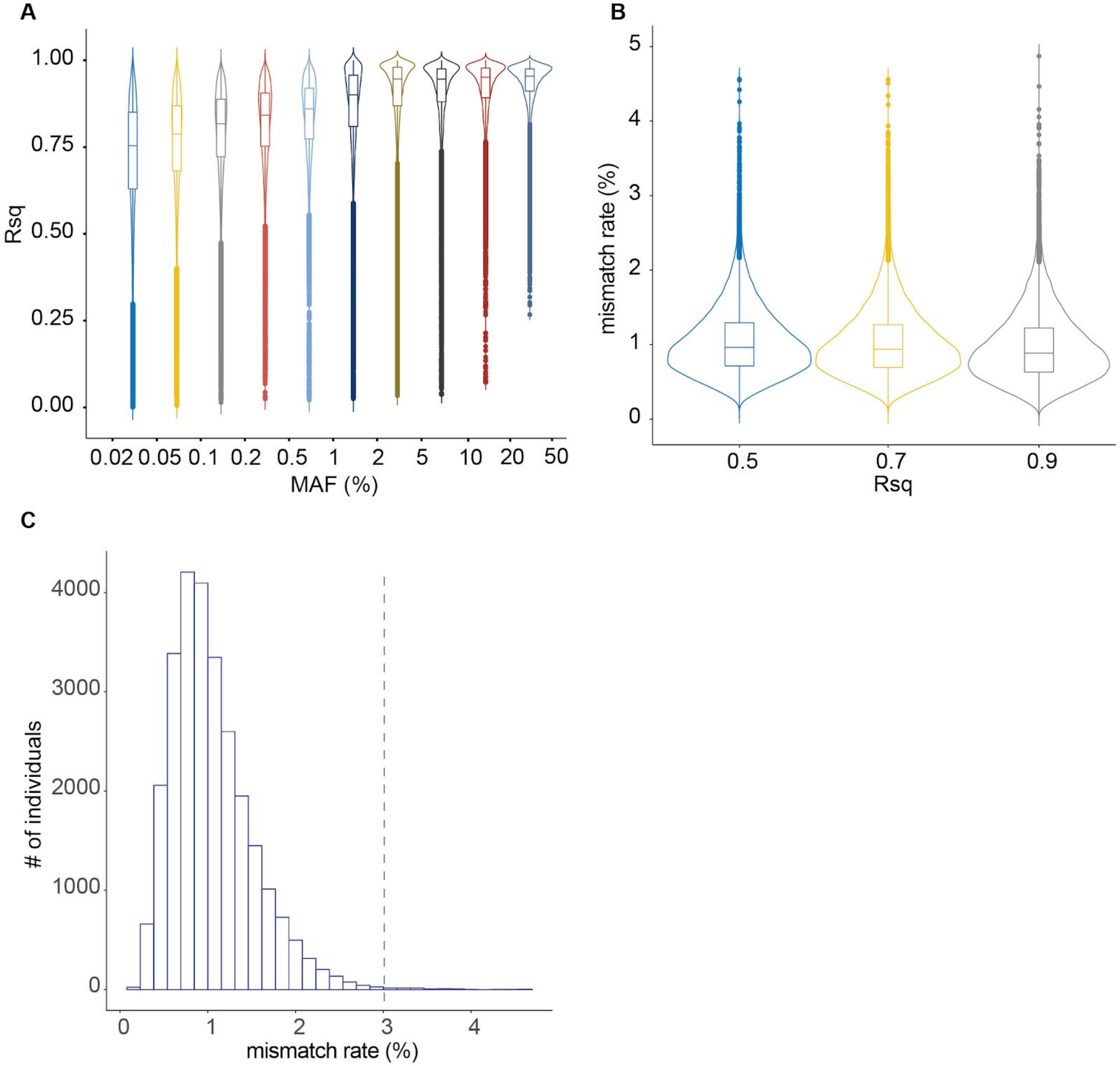

Supplementary Figure 4| MDS plots

MDS plots of the first two components. MDS components for all HapMap population and SPARK trio children were analyzed jointly and plotted separately (HapMap population on the left and SPARK trio children on the right panel). The area enclosed in a gray box indicates sub-populations that were estimated by the representative HapMap population. Subpopulations were defined as 5 standard deviation (S.D.) around the centroid of the CEU and TSI for European [EUR], CHB, CHD and JPT for EastAsian [EAS], and YRI and LWK for African [AFR]. See also Supplementary Table 2.

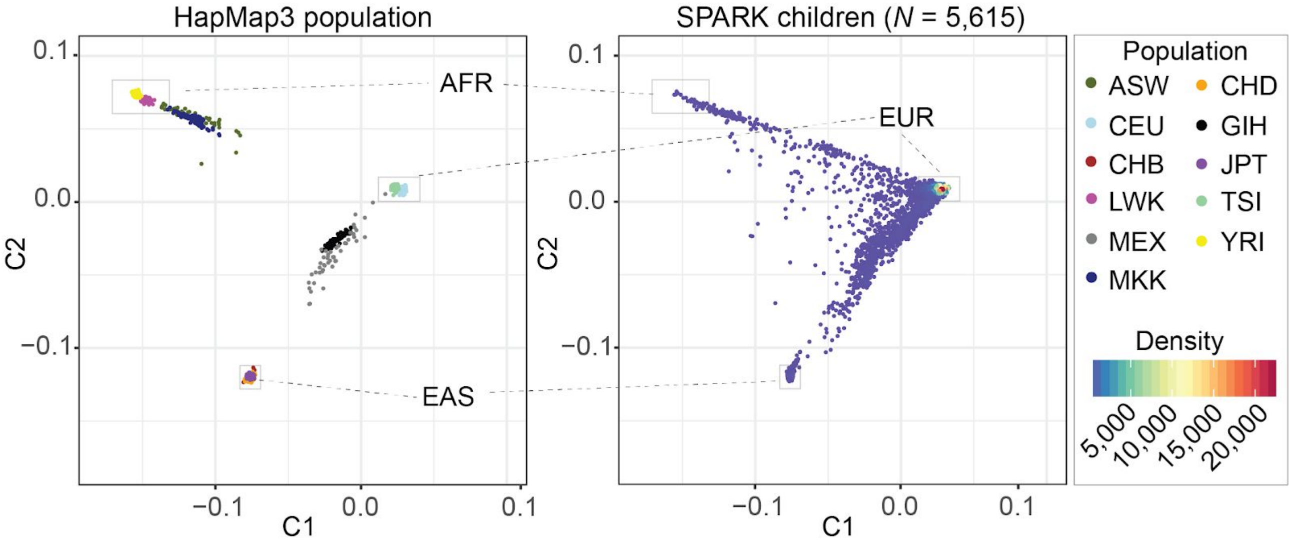

Supplementary Figure 5| QQ plots for SPARK studies

QQ-plots represent P-value distributions as observed against those expected under the null for (**A**) SPARK full data set and (**B**) SPARK data set subset to families of European ancestry.

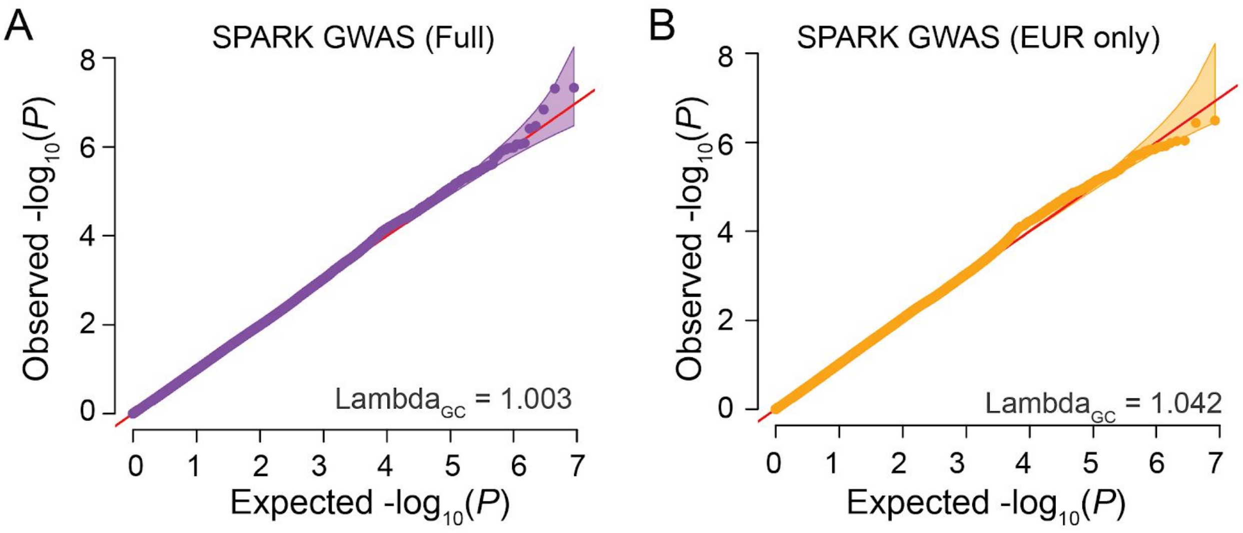

Supplementary Figure 6| Forest plots for the index SNPs identified in the SPARK full dataset and iPSYCH+PGC meta-analysis

SNP was indicated as *Chromosome*:*Position*:*Effect Allele*:*Non-Effect Allele_SNPID*.
N denotes the total number of individuals including pseudocontrols used for association study.

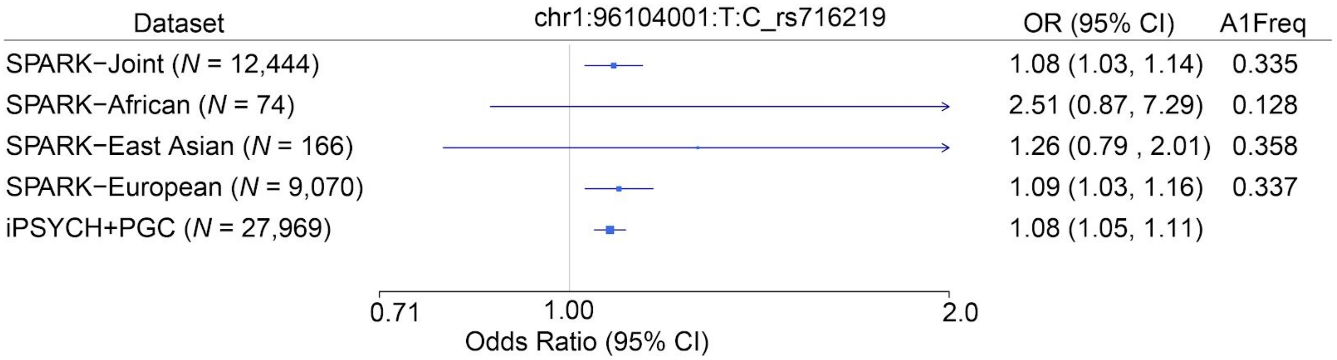

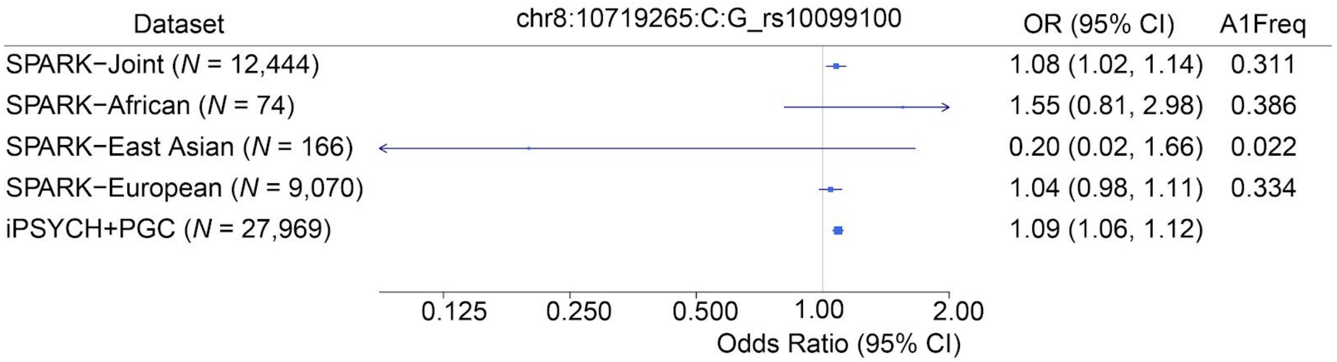

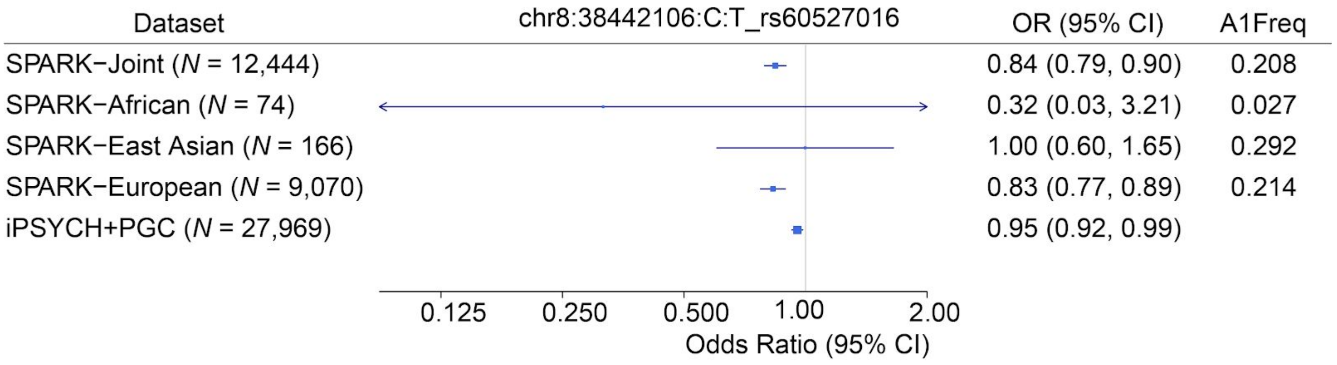

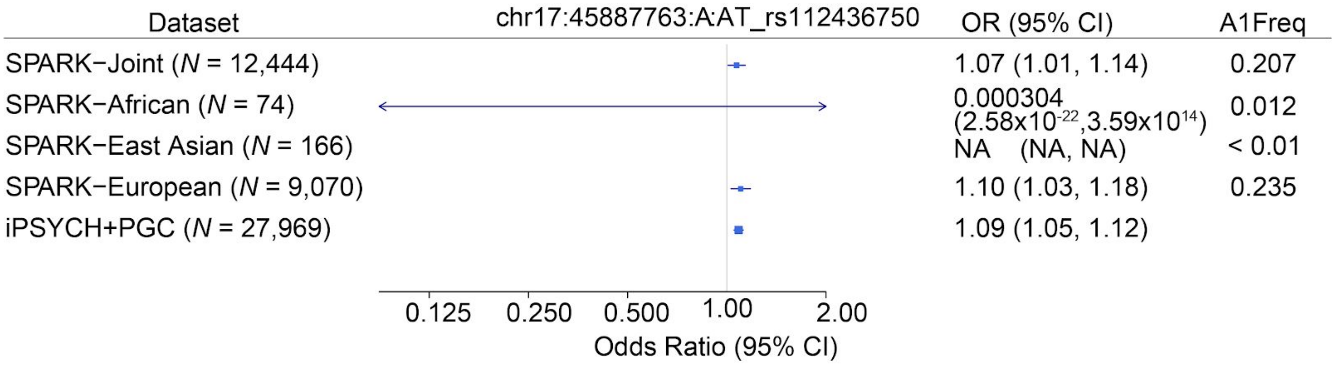
Dataset
chr17:45887763:A:AT_rs112436750
OR (95% CI)
A1Freq
SPARK−Joint (N = 12,444)
1.07 (1.01, 1.14)
0.207
SPARK−African (N = 74)
0.000304 (2.58x10-22,3.59x1014)
0.012
SPARK−East Asian (N = 166)
NA (NA, NA)
< 0.01
SPARK−European (N = 9,070)
1.10 (1.03, 1.18)
0.235
iPSYCH+PGC (N = 27,969)
1.09 (1.05, 1.12)
0.125
0.250
0.500
1.00
2.00
Odds Ratio (95% CI)

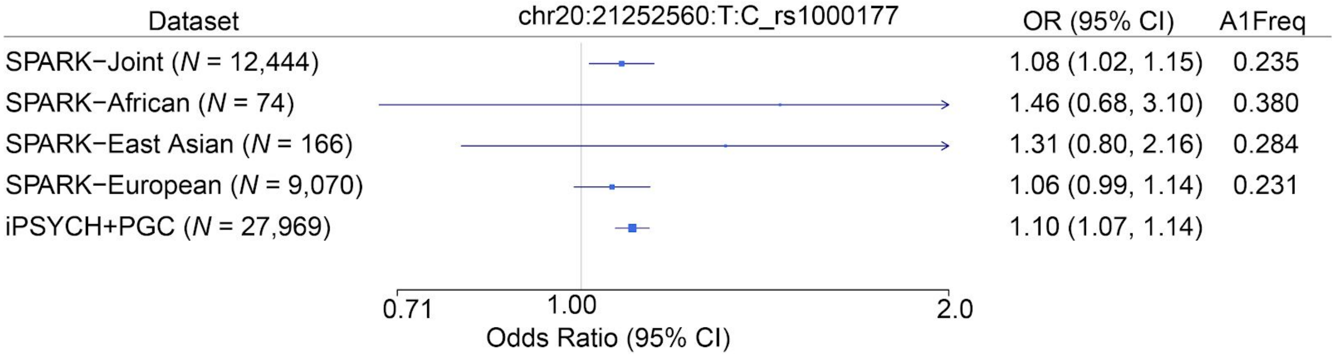
Dataset
chr20:21252560:T:C_rs1000177
OR (95% CI)
A1Freq
SPARK−Joint (N = 12,444)
1.08 (1.02, 1.15)
0.235
SPARK−African (N = 74)
1.46 (0.68, 3.10)
0.380
SPARK−East Asian (N = 166)
1.31 (0.80, 2.16)
0.284
SPARK−European (N = 9,070)
1.06 (0.99, 1.14)
0.231
iPSYCH+PGC (N = 27,969)
1.10 (1.07, 1.14)
0.71
1.00
2.0
Odds Ratio (95% CI)

Supplementary Figure 7| Regional association plot for the index SNP (rs716219) on Chromosome 1 from the meta-analysis (EUR)

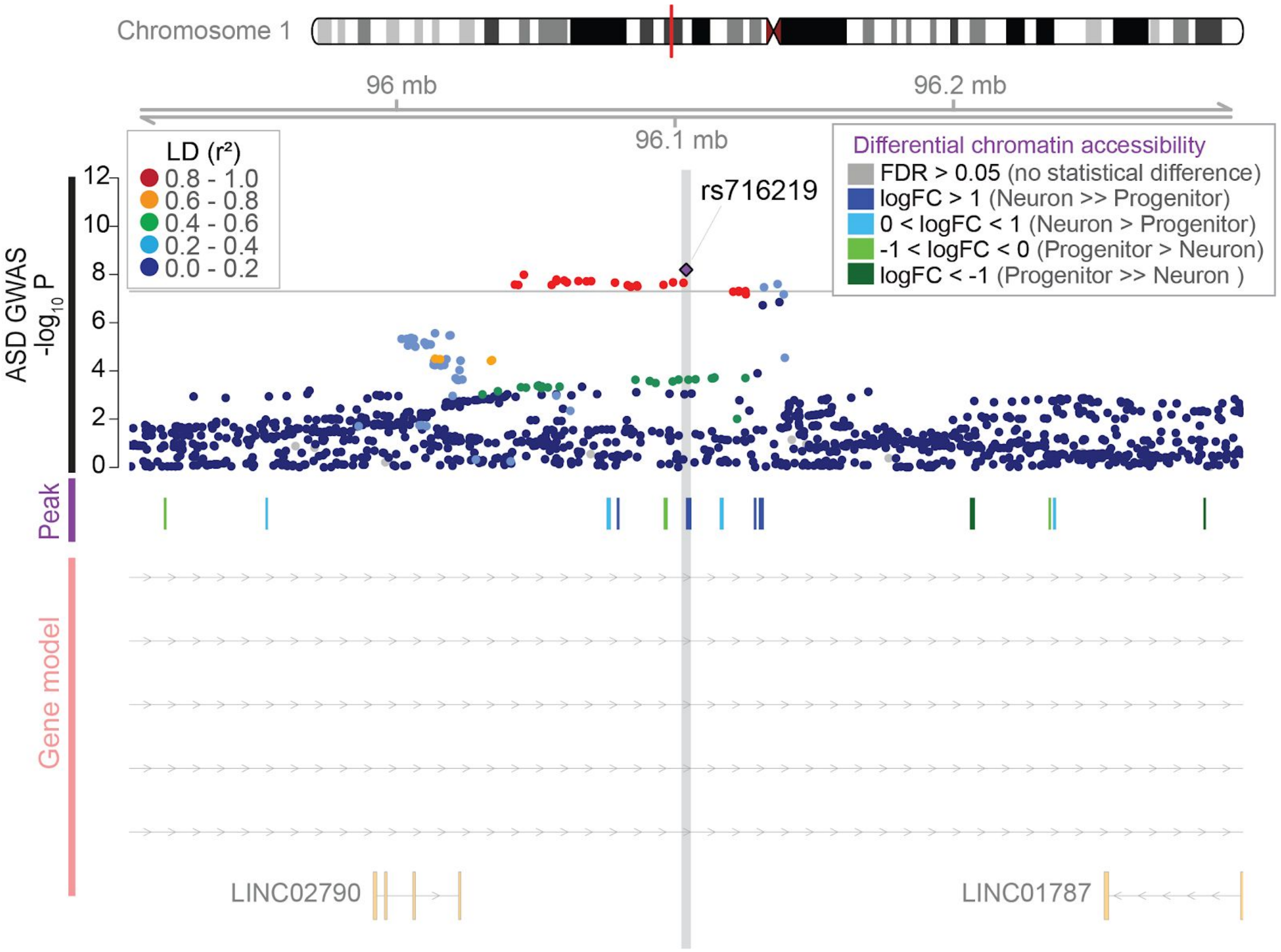

Supplementary Figure 8| Regional association plot for the index SNP (rs10099100) on Chromosome 8 from the meta-analysis (EUR)

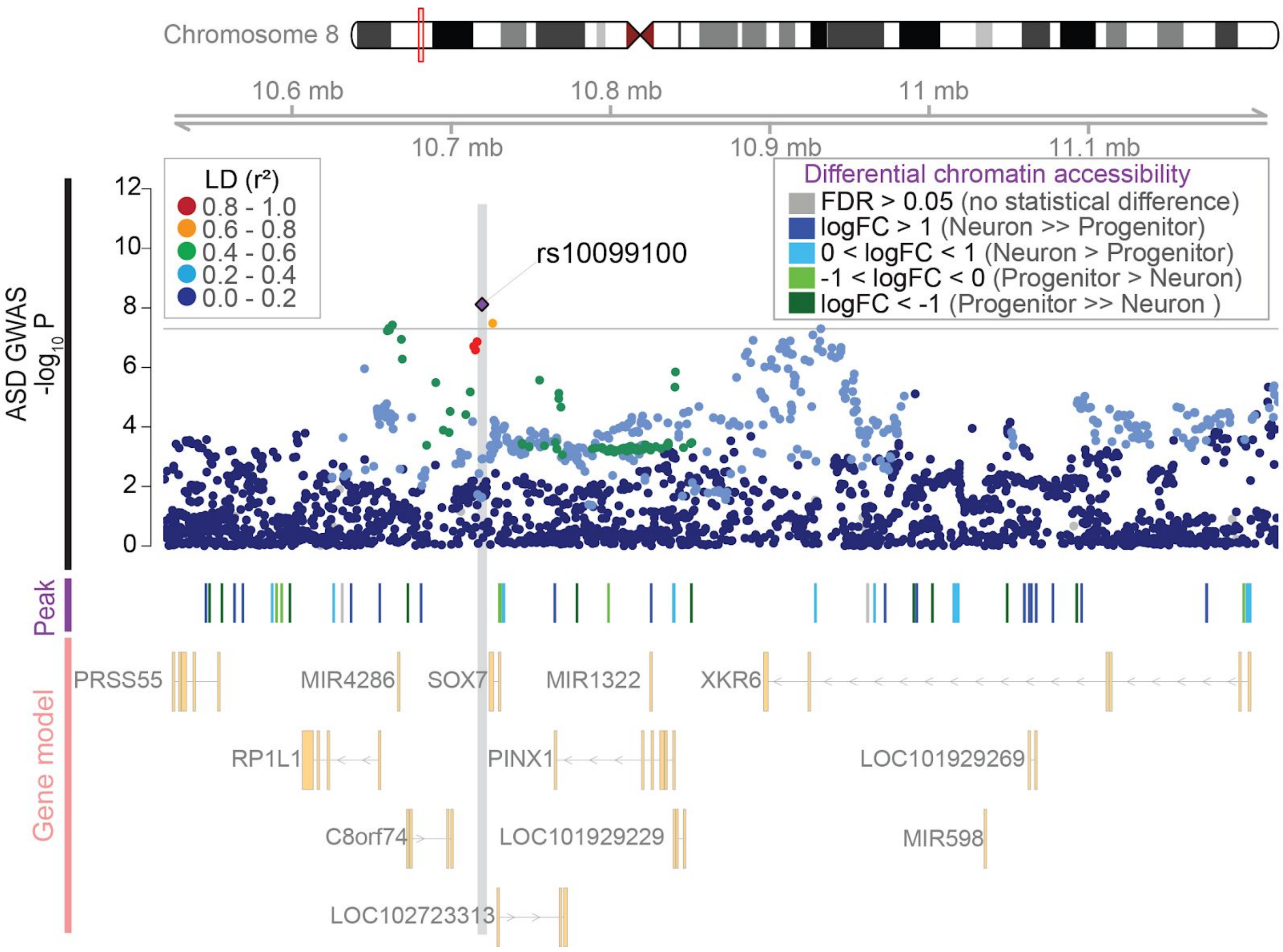

Supplementary Figure 9| Regional association plot for the index SNP (rs112436750) on Chromosome 17 from the meta-analysis (EUR)

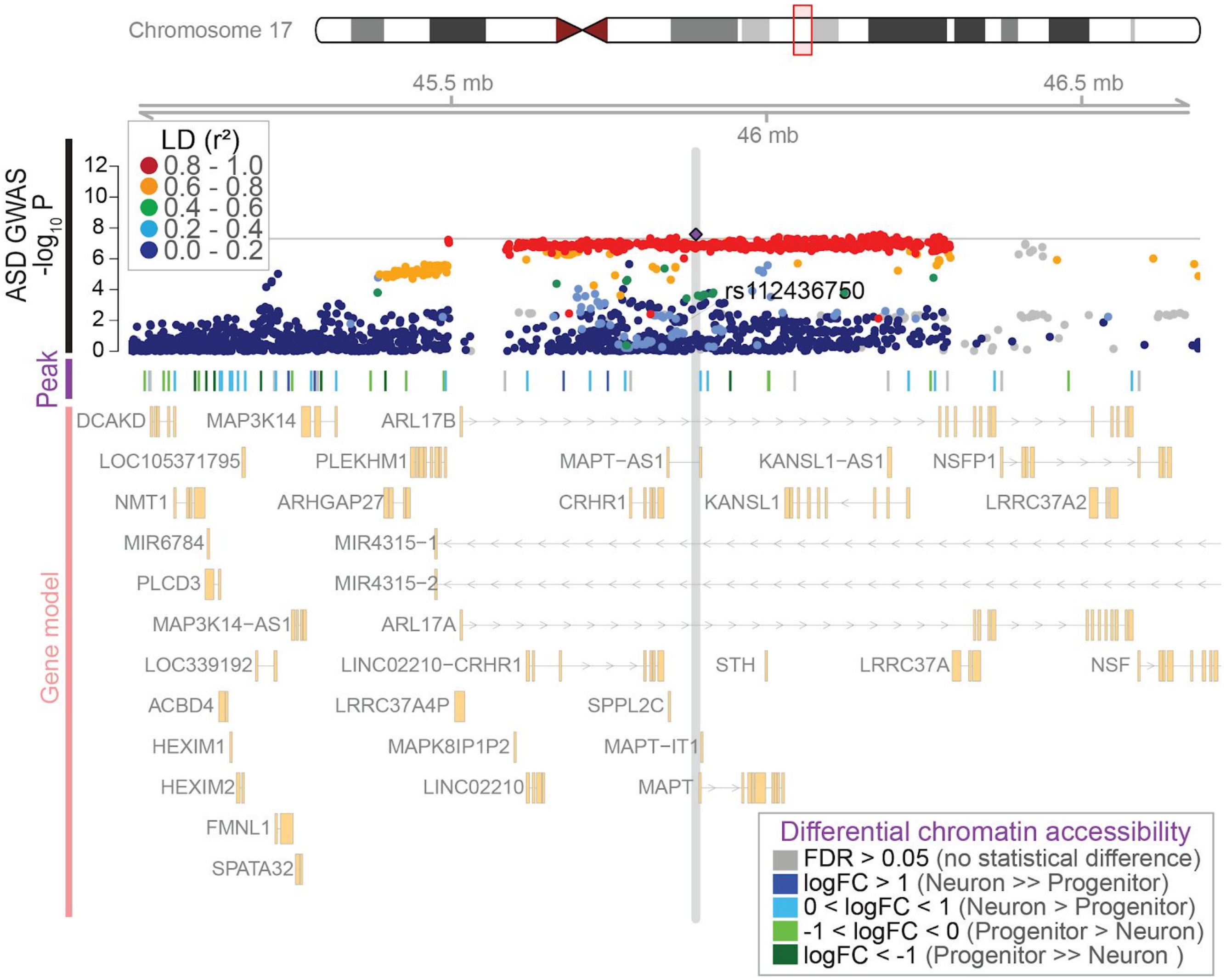

Supplementary Figure 10| Regional association plot for the index SNP (rs1000177) on Chromosome 20 from the meta-analysis (EUR)

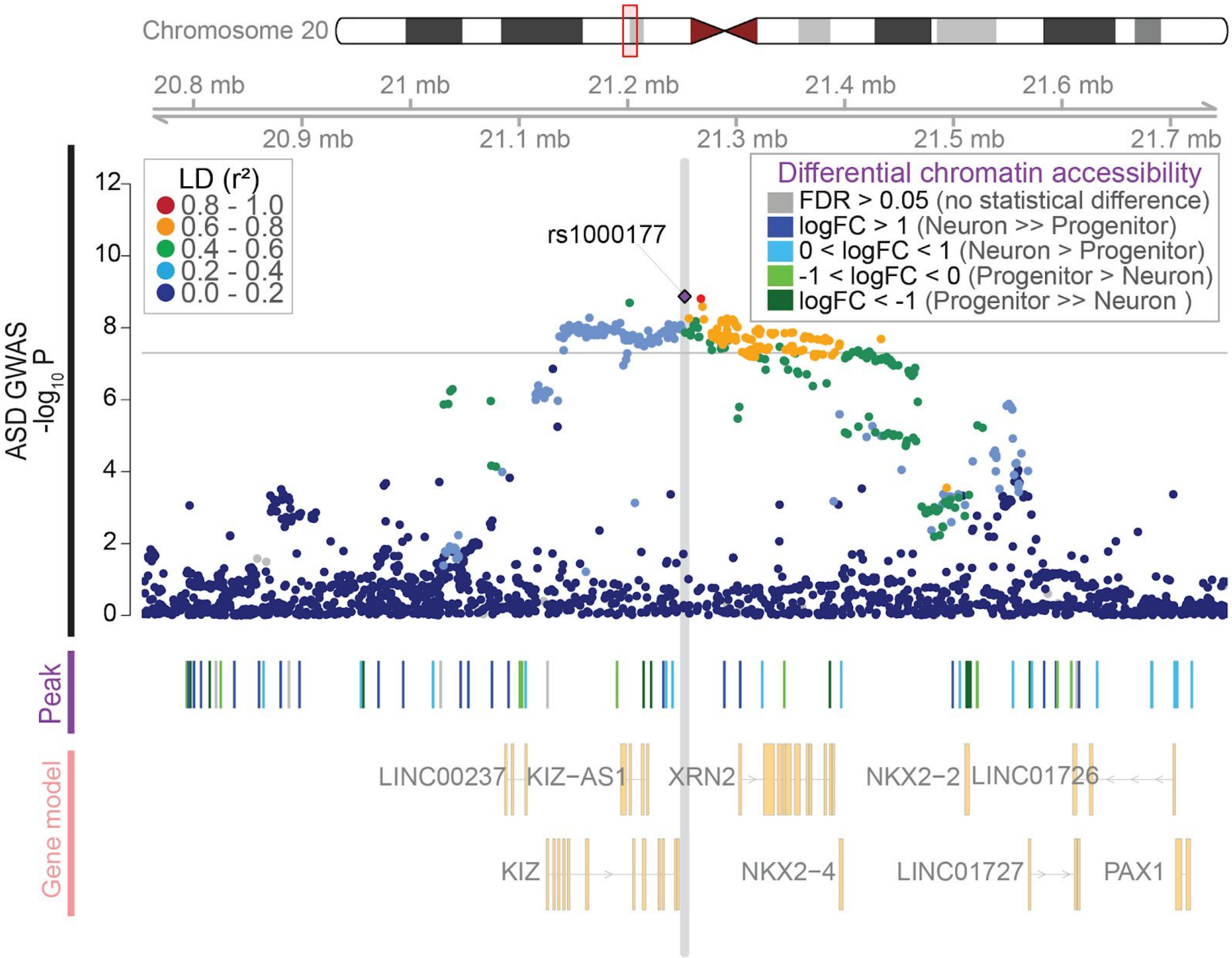

Supplementary Figure 11| Variance explained (Nagelkerke R^2) by the ASD PRS

The depth of color indicates the log fold change (logFC) for ASD cases and pseudo-controls and the number at the top of each bar indicates significance. The x-axis indicates the GWAS P-value thresholds used to group SNPs from iPSYCH-PGC[1]. The y-axis refers to the percentage of variance as Nagelkerke R^2.

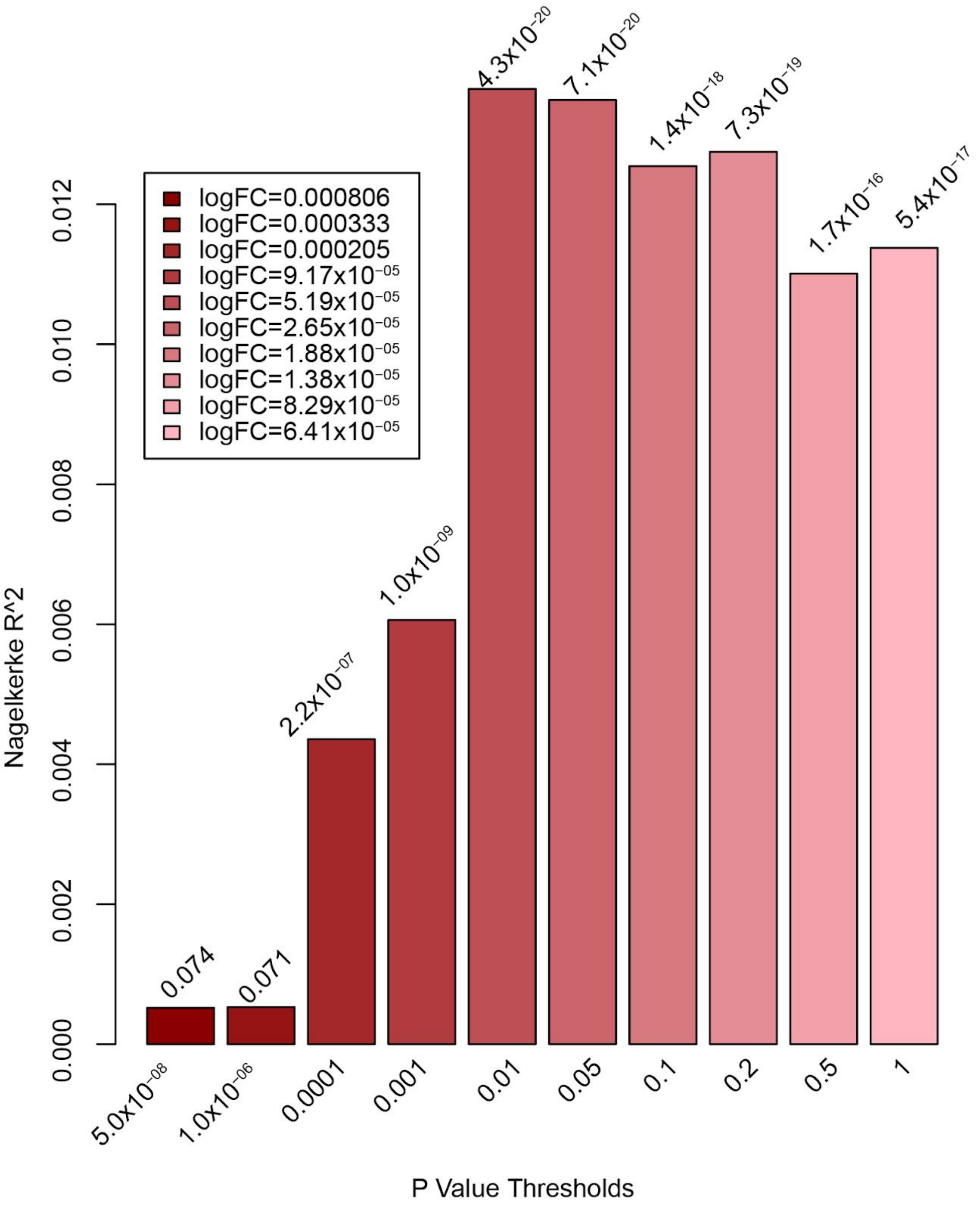

Supplementary Figure 12| Partitioned heritability enrichment of tissues implicates cortical development in ASD risk

(A) Heritability enrichment in active enhancer and promoter regions present in different tissues shows the critical role of developing the brain in ASD etiology. **(B)** Heritability enrichment in differential chromatin accessibility from the developing fetal cortical wall. (upper) The x-axis represents tissue types and the y-axis indicates heritability enrichment. The error bar shows a 95% confidence interval. (lower) The x-axis represents tissue types and the y-axis indicates statistical significance as -log10(FDR). BRN: Brain, ADRL: Adrenal, BLD: Blood, BRST: Breast, CRVX: Cervix, ESDR: ESC_derived, GI: GI_duodenum, GI_colon, GI_rectum, GI_stomach, GI_intestine, GI_colon, GI_rectum, GI_duodenum and GI_esophagus, HRT: Heart, KID: Kideney, LIV: Liver, LNG: Lung, MUS: Muscle, OVRY: Ovary, PANC: Pancreas, PLCNT: Placenta, SPLN: Spleen, STRM: Stromal connective, THYM: Thymus, VAS: Vascular, CP: Peaks more accessible in cortical plate, GZ: Peaks more accessible in germinal zone. * FDR < 0.05, ** FDR < 0.01.

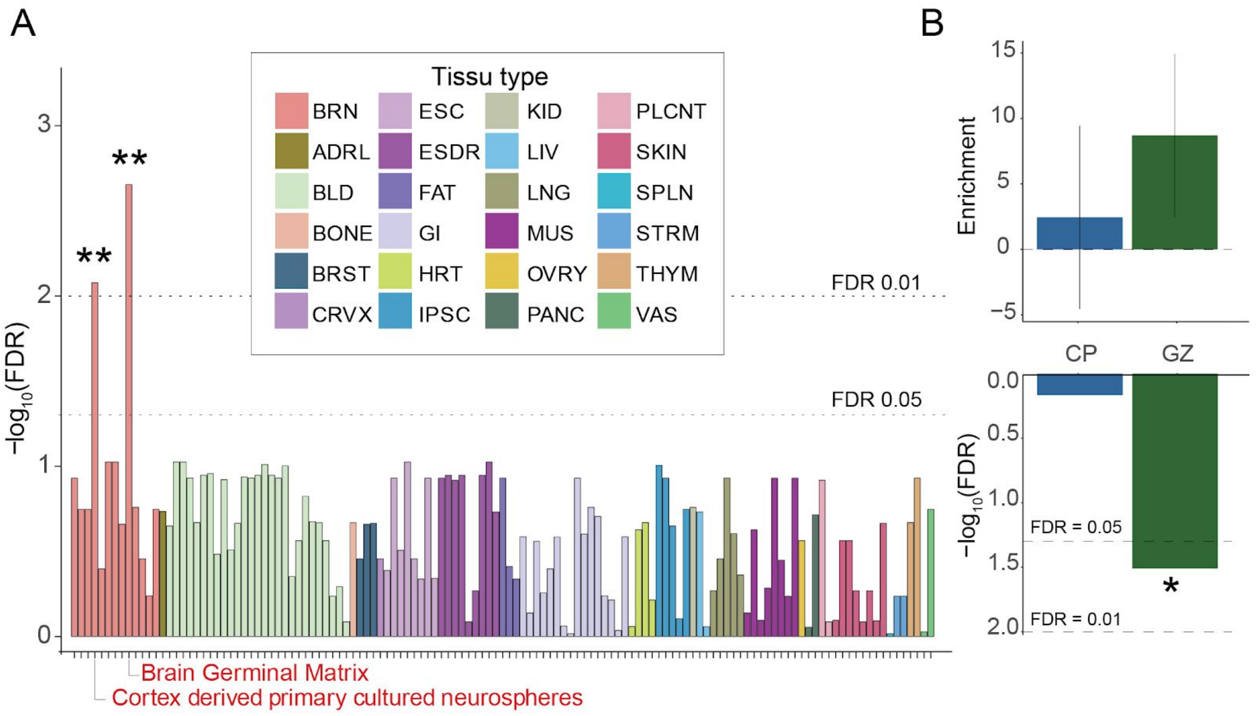

Supplementary Figure 13| Profiling tissue type specific H-MAGMA gene

(**A**) A Gene-based association result from H-MAGMA using Hi-C interaction in the adult brain[2]. The x-axis indicates the start position of genes (hg19). (**B**) Overlap of H-MAGMA genes based on fetal and adult brain Hi-C data. 124 genes including *FOXP2* and *BCL11A* were only identified in fetal-tissue based H-MAGMA. (**C**) Gene ontologies enriched for ASD linked genes from the fetal brain (left) and adult brain (right) H-MAGMA results. Top 20 GO from each dataset was shown.

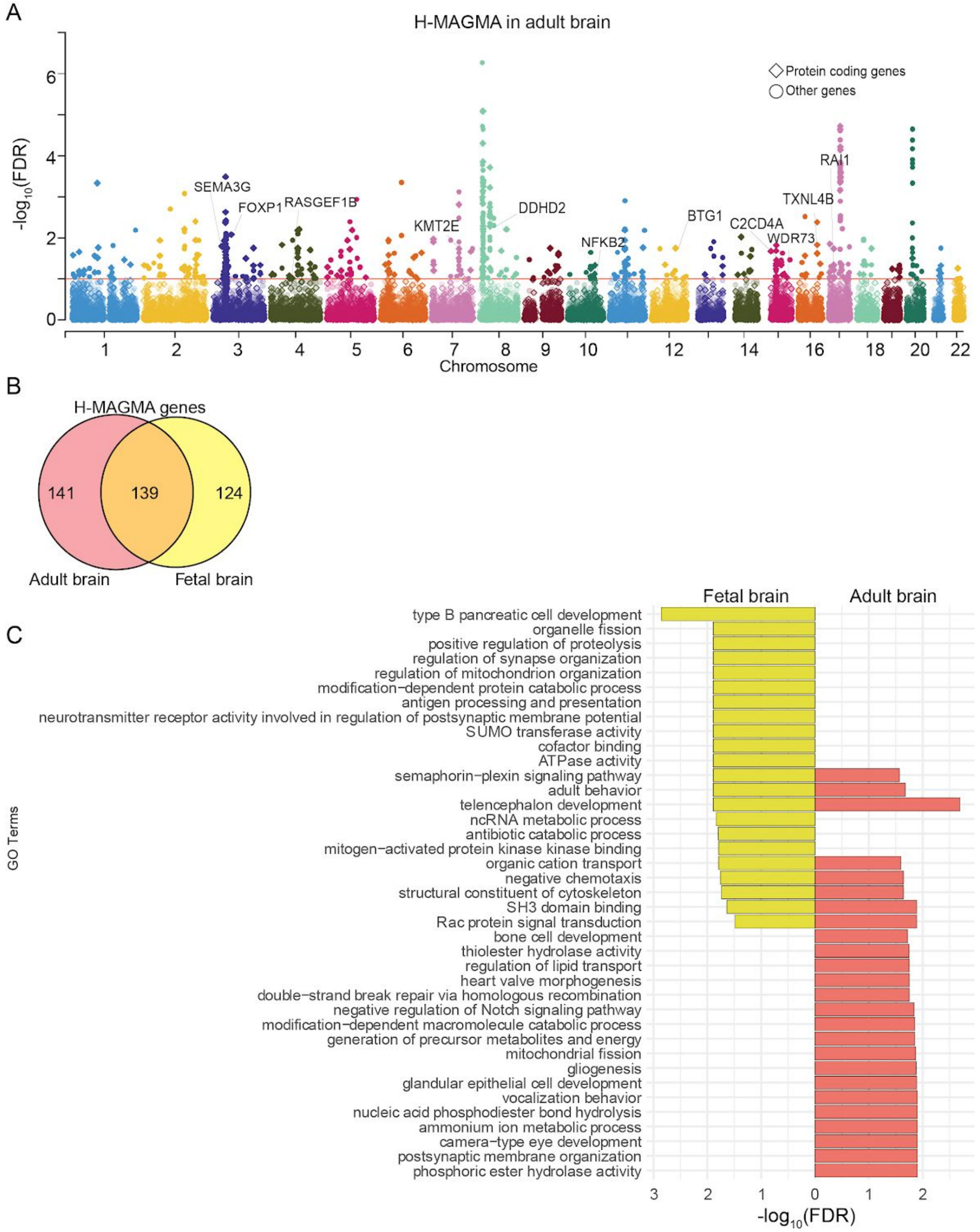

Supplementary Figure 14| Developmental trajectory of ASD risk genes

(**A**) Normalized expression values of ASD H-MAGMA genes in the cortex across different developmental time points (see more details in Supplementary Methods). Each point denotes mean cortical expression level of ASD H-MAGMA genes at a given age. LOESS smooth curve plotted with individual data points (N=410 and 453 for prenatal and postnatal samples, respectively). (**B**) Three gene clusters (modules) were identified by WGCNA. Most genes (50.45%) were in the module 'MEturquoise'. (**C**) Using the same strategy as in (**A**), mean expression level of genes in each module were plotted. Genes in 'MEturquoise' were highly expressed in early development similar to the (**A**).

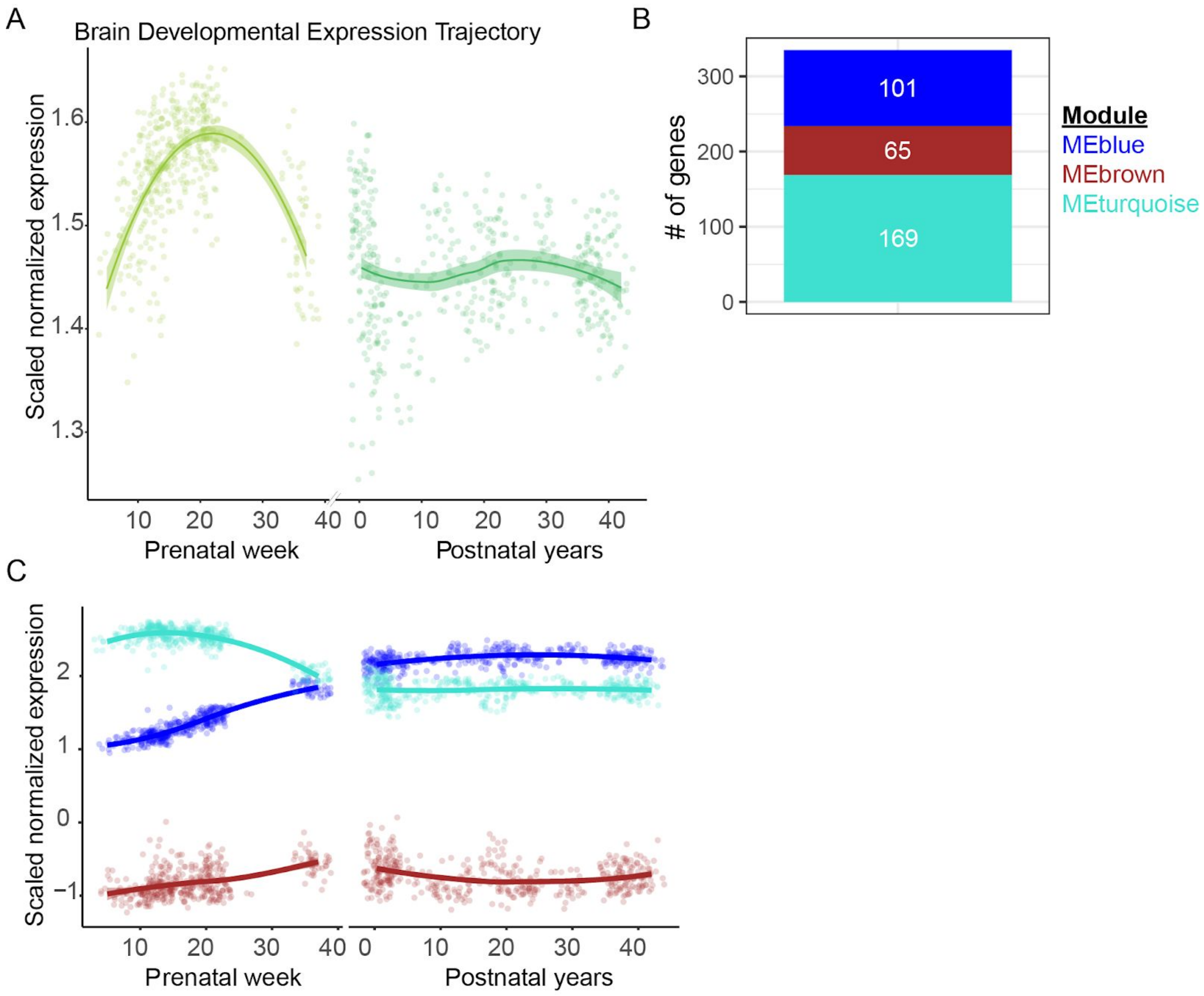

Supplementary Figure 15| Comparison of credible SNPs from Schizophrenia (SZ) and ASD

(**A**) Association signals from ASD GWAS (SPARK dataset) (upper) and SZ GWAS[3] (bottom). (**B**) Overlapped credible SNPs between ASD GWAS and SZ GWAS. (**C**) Direction of effects on schizophrenia or ASD risk are consistent at the locus. (**D**) Posterior probability distribution of shared credible SNPs is significantly higher than that of ASD-only credible SNPs. (**E**) Statistical significance of GWAS result for credible SNPs. P-values were obtained by the Wilcoxon test.

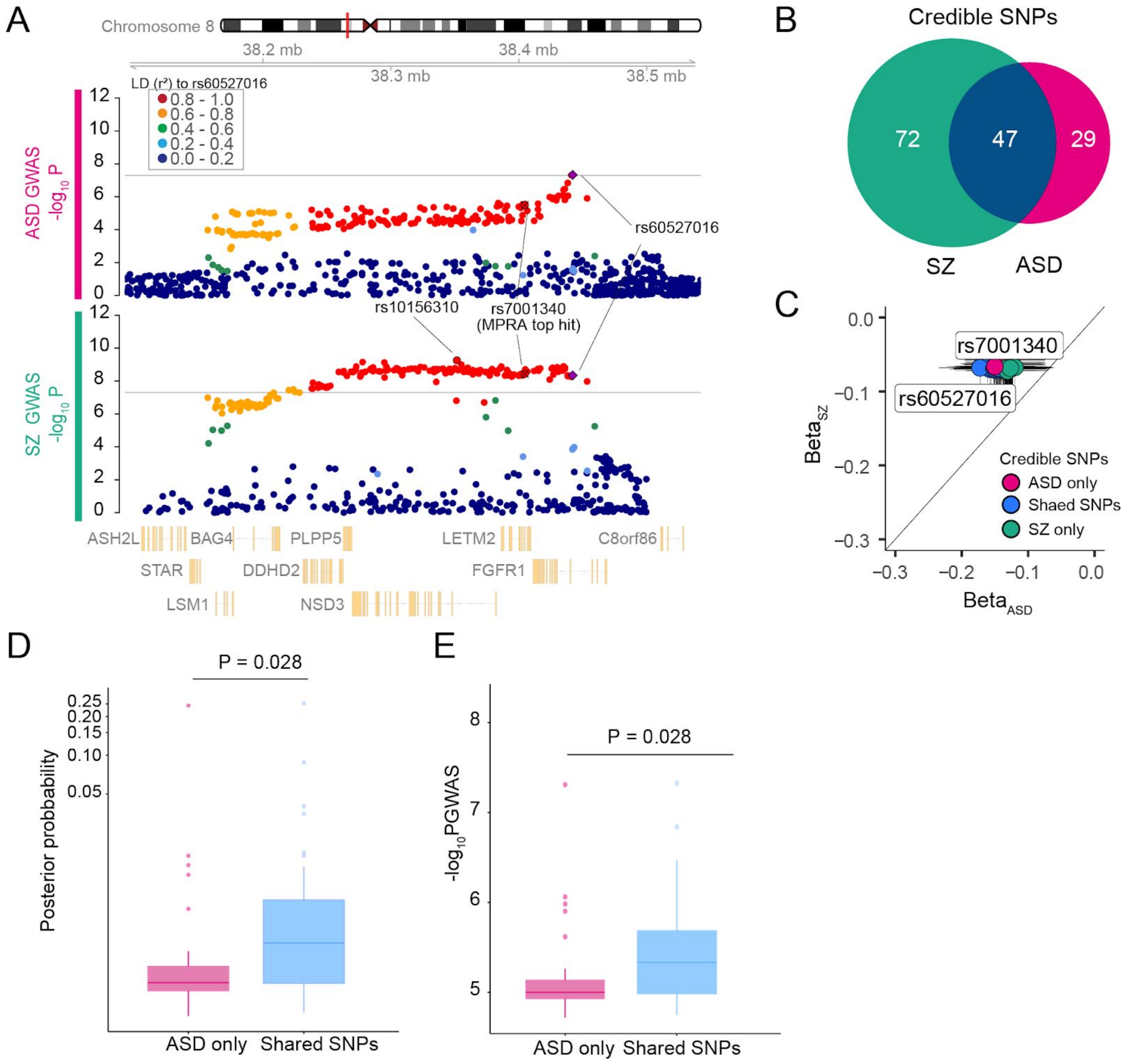

Supplementary Figure 16| Quality check for MPRA experiment

(**A**) Barcode coverage across 98 tested variants. (**B**) Reproducibility between individual biological replicates. Each dot denotes log(sum of barcoded RNA counts/sum of barcoded DNA counts) for each variant. R indicates correlation coefficients from generalized linear regression. (**C**) Volcano plot of 98 variants within the novel loci identified in SPARK GWAS (chr8:38.19M - chr8:38.45M) showing fold change of barcoded expression of risk alleles compared to protective alleles in the x-axis, and statistical significance (-log10(FDR)) in the y-axis. The vertical lines indicate the 1.5 or -1.5 log FC thresholds. (**D**) Distribution of barcode GC content mapped to rs7001340 alleles. P-value calculated by a two-sample t-test.

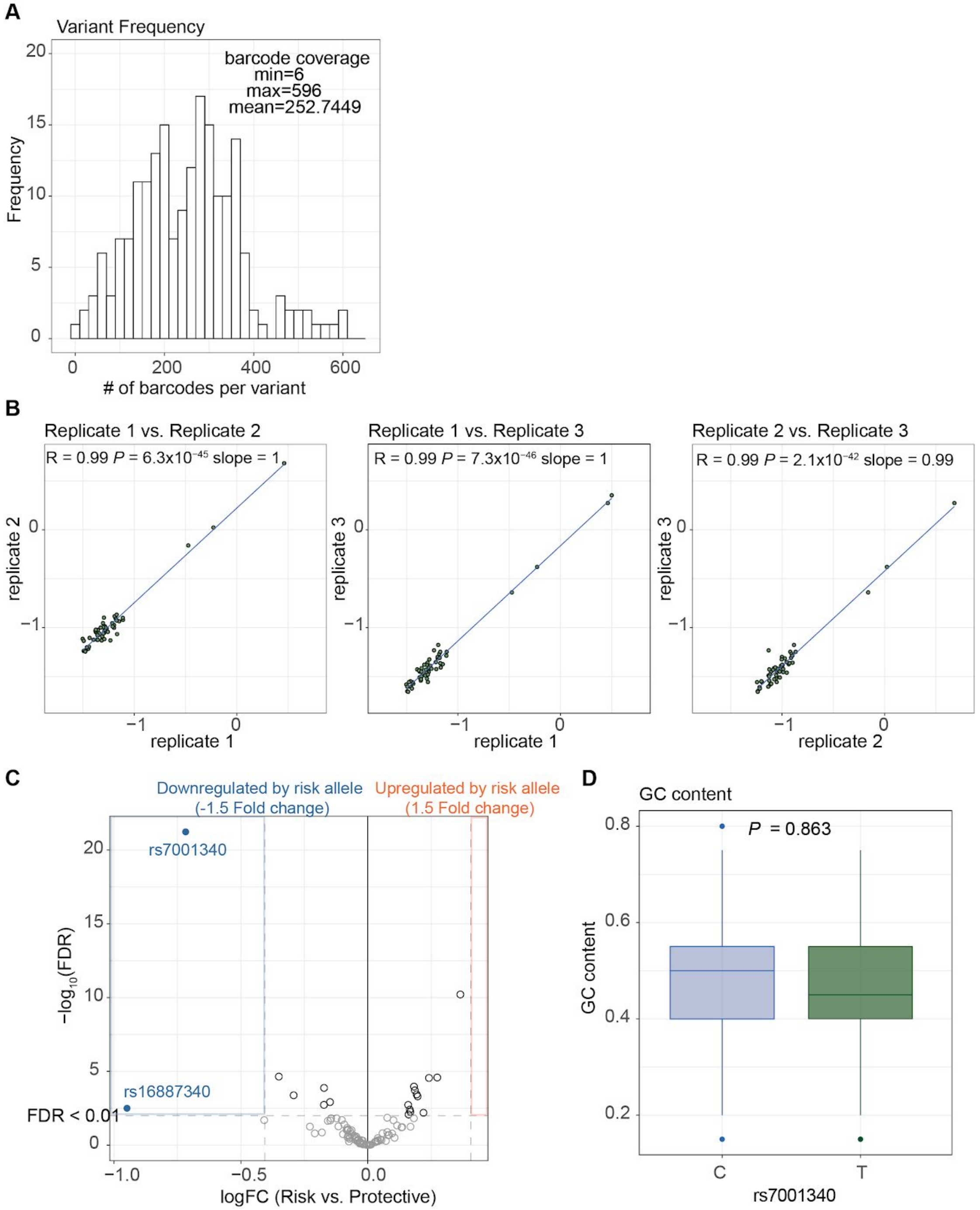

Supplementary Figure 17| Conserved chromatin structure in neural progenitors and HEK cells

Chromatin accessibility at the DDHD2 locus. From top to bottom, the panels show 1) SNP - ASD associations in SPARK GWAS colored by LD structure to the putative causal variant (rs7001340), 2) MPRA based allelic associations assessed in HEK293T cells (GSM1008573)[4], 3) and 4) ATAC-Seq read depth from neuronal cells and neural progenitor cells, 5) differential chromatin accessibility between neuronal cells and neural progenitor cells related to panel 3) and 4). 5) chromatin accessibility in HEK 293T cell colored by peak score. 6) Gene models.

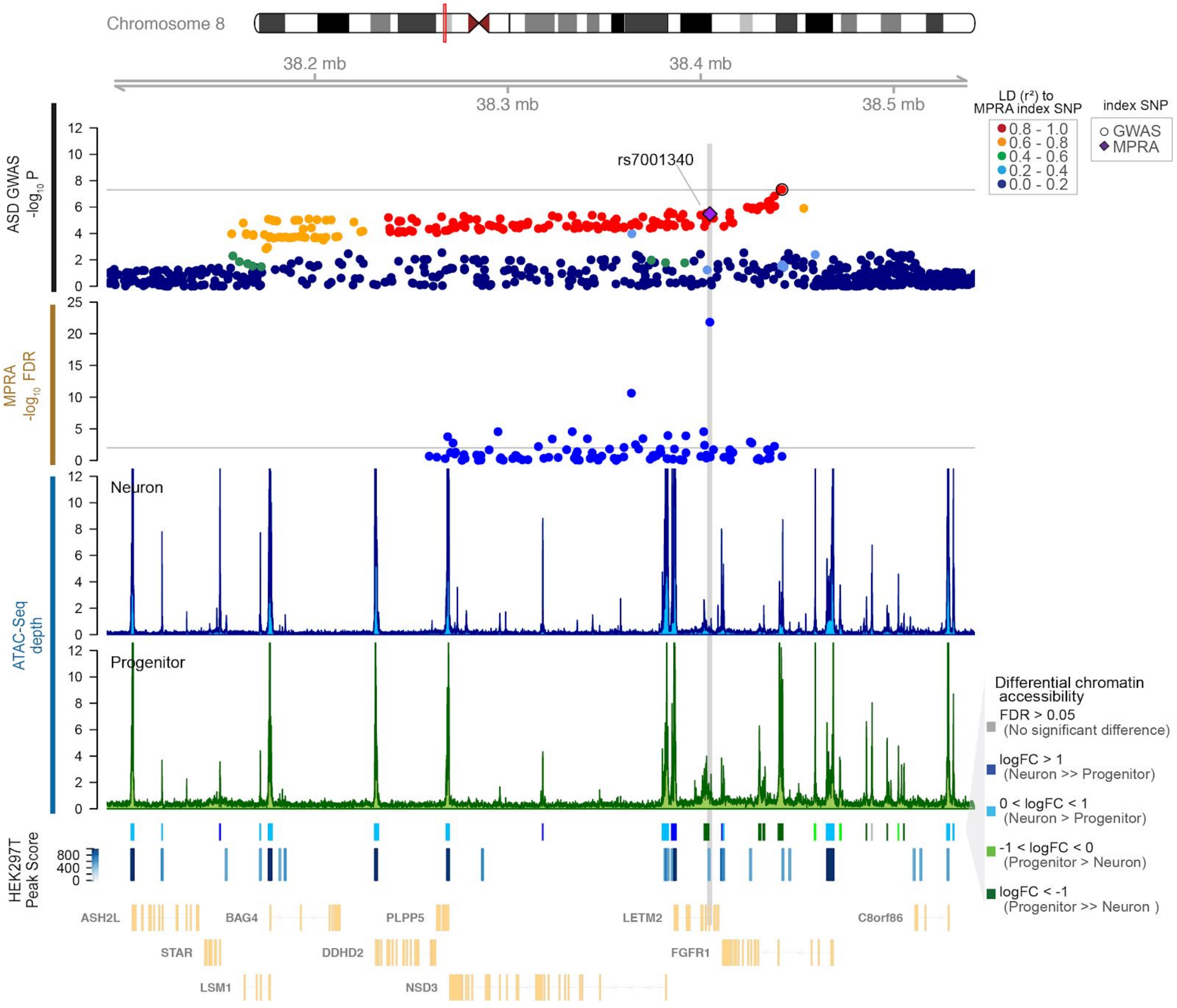

Supplementary Figure 18| Disruption of transcription factor binding motifs by rs7001340

(**A**) The rs7001340 T allele is predicted to disrupt TBX1 (left) and SMARCC1 (right) binding motif[5,6]. The position of rs7001340 is marked as a red number on the x-axis and highlighted in grey. (**B**) Heatmap for expression of transcription factors in brain cell types from scRNA-seq. Color indicates normalized expression for given cell types. scRNA-seq data were obtained from http://solo.bmap.ucla.edu/shiny/webapp/ [7]

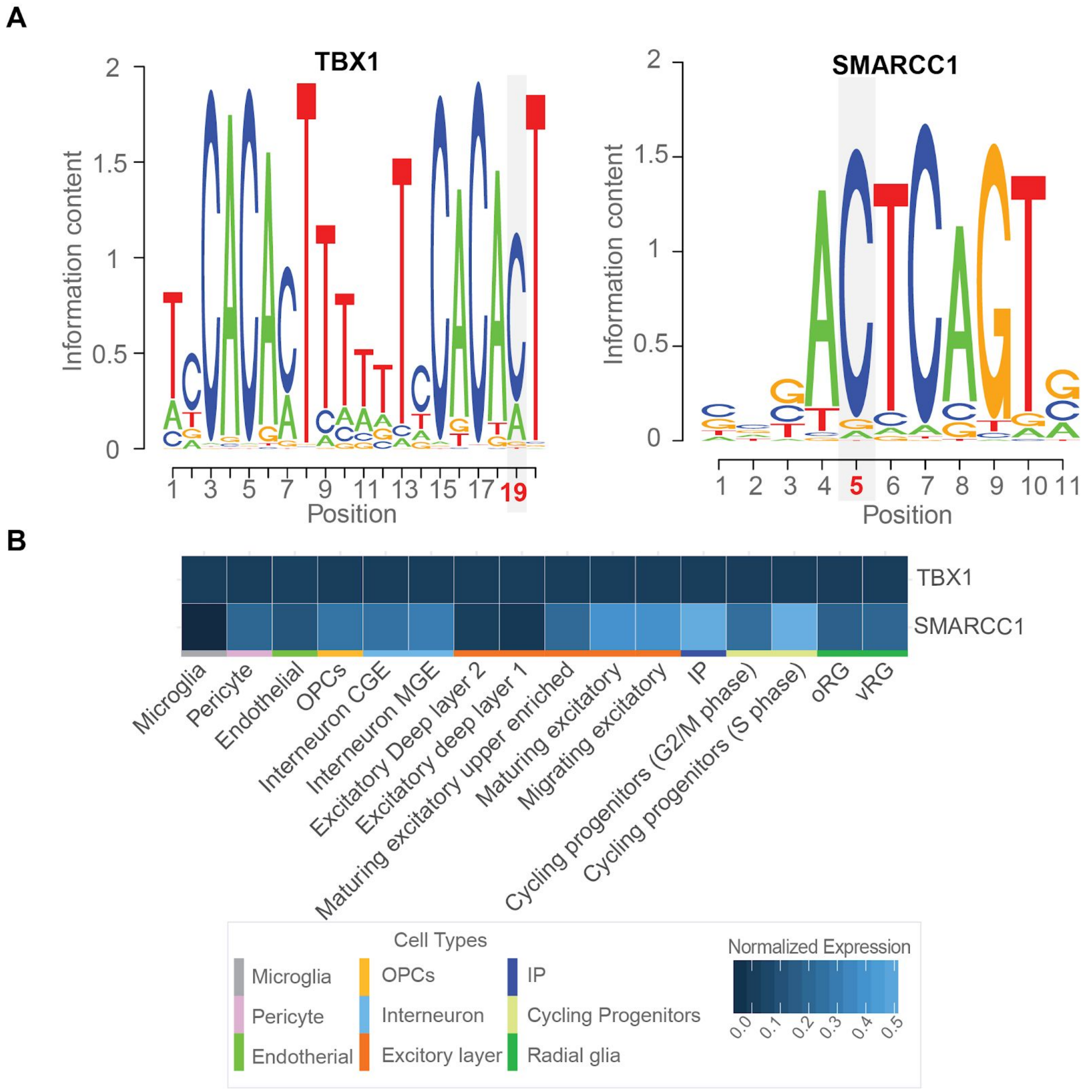

Supplementary Figure 19| Expression level of eGenes regulated by rs7001340

Expression levels of (**A**) *LETM2* and (**B**) *LSM1* by rs7001340 genotypes in the adult brain. The ASD risk allele for rs7001340 is T and the protective allele is C. Individuals with allelic dosage (0-0.1 as C/C, 0.9-1.1 as C/T, 1.9-2.0 as T/T) are shown.

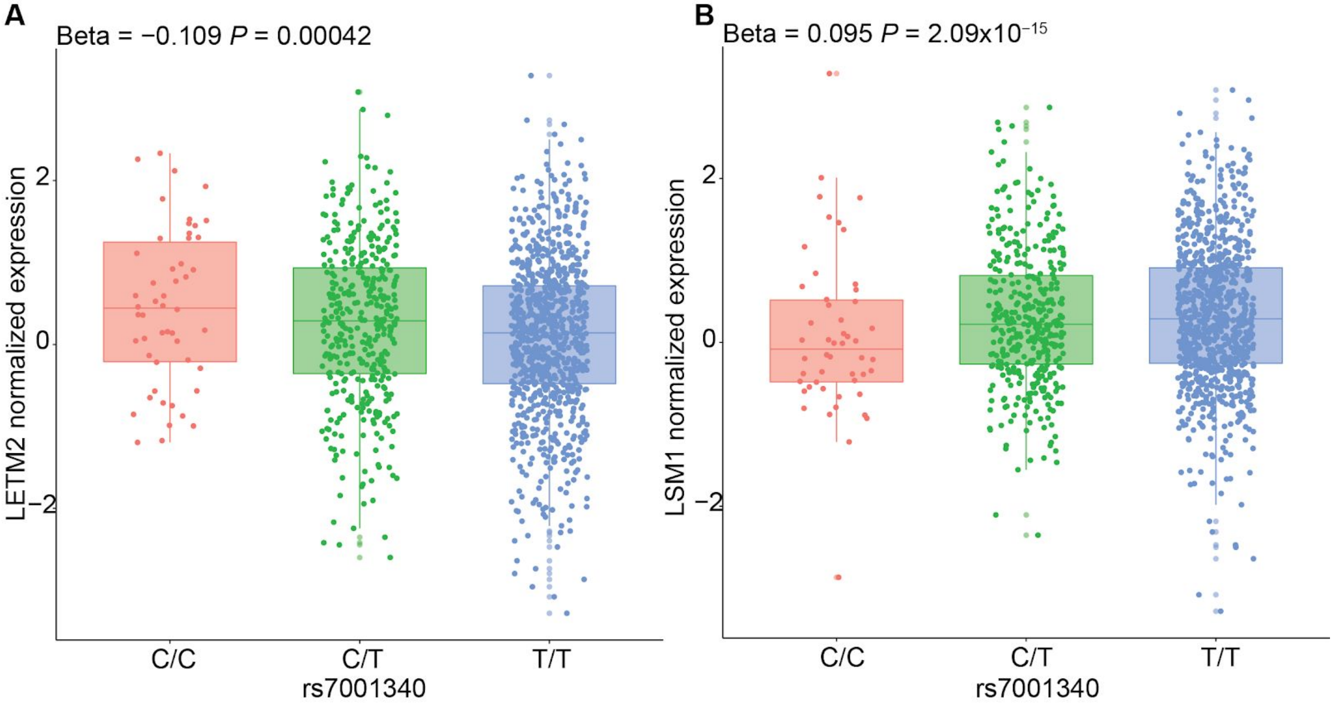

Supplementary Figure 20| Alternative approaches to test DDHD2 association in multiple tissue types

The scatter plots compare per-gene -log10(P) obtained by H-MAGMA (fetal brain) (x-axis) and PrediXcan or FUSION (y-axis in (**A**) and (**B**), respectively) in dorsolateral prefrontal cortex (DLPFC) from CommonMind Consortium, adipose tissue from GTEx or whole blood tissues from GTEx. (**C**) The top panel indicates genes in the locus including *DDHD2* predicted by TWAS (in green), and manhattan plot in the bottom indicates ASD GWAS signal before (gray) or after (blue) conditioning on the TWAS predicted expression level of *DDHD2*, demonstrating that GWAS signal at this region is explained mainly by predicted expression of DDHD2 .

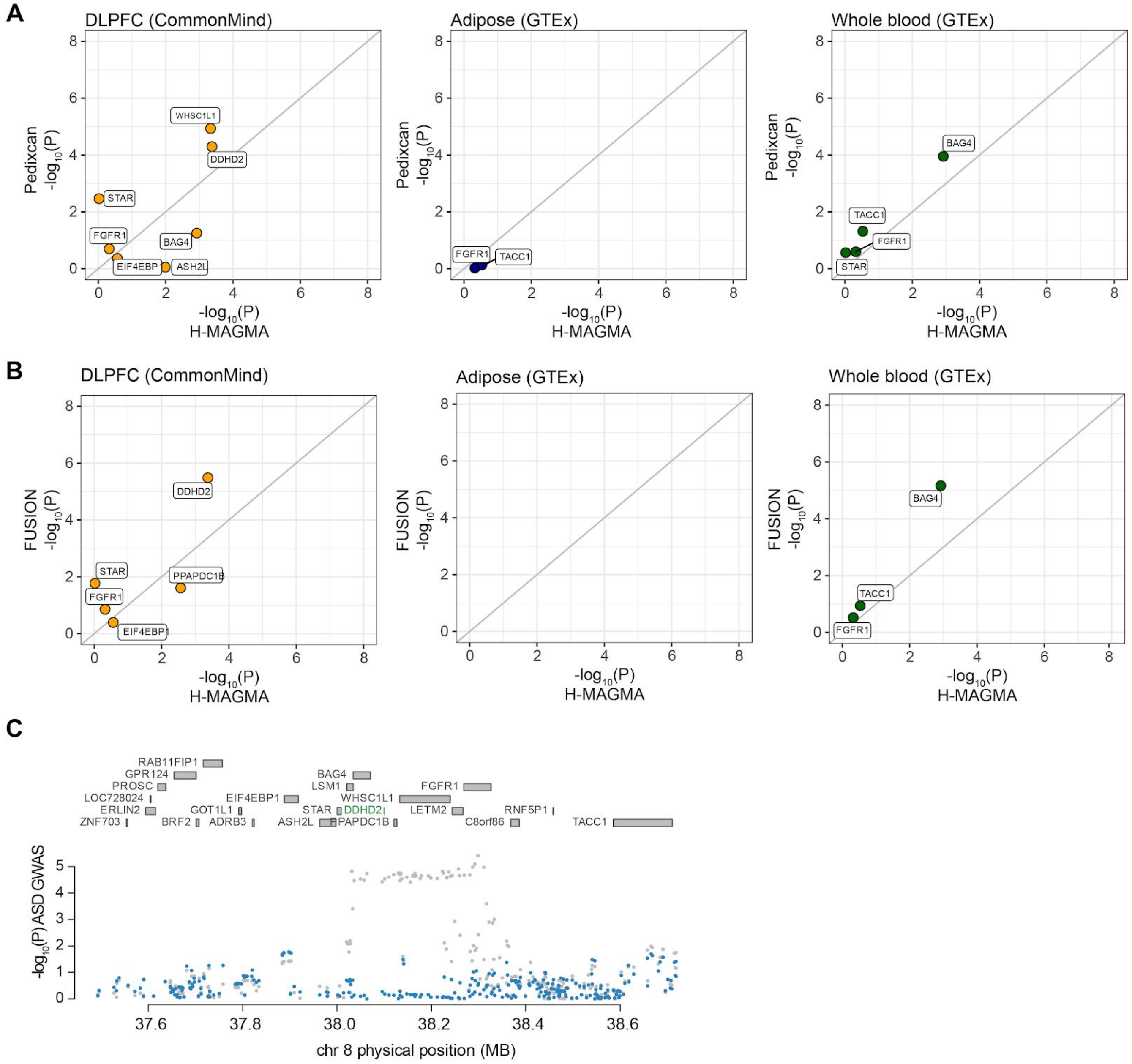

Supplementary Tables

Supplementary Table 1| Study characteristics

	GWAS (% male)	PRS[1] (% male)
**DSM Sub-diagnosis(if applicable)**		
Asperger's Disorder	613 (80.10%)	463 (82.07%)
Autism or Autistic Disorder	728 (81.59%)	469 (81.88%)
Autism Spectrum Disorder	4,407 (79.26%)	2,845 (78.70%)
NOS	440 (80.00%)	317 (81.07%)
**Ancestry based on genotypes**		
European	4,535 (78.77%)	4,097 (79.62%)
African	37 (75.68%)	-
East Asian	83 (87.95%)	-
Others / Admixed	1,567 (81.80%)	-
Total	6,222 (79.65%)	4,097 (79.62%)
**Family Type**		
Multiple-affected children w/ an affected parent	-	14 (71.43%)
Multiple-affected children w/ unaffected parents	-	420 (78.10%)
One affected child w/ affected parent(s)	-	45 (88.89%)
One affected child w/ unaffected parents	-	3,618 (79.71%)
Total		4,097 (79.62%)

[1] for PRS, we analyzed one individual from each family from European ancestry (4,097)

NOS : Pervasive Developmental Disorder - Not Otherwise Specified

Supplementary Table 2| HapMap population used for MDS

This table is related to Supplementary Figure 2

ID	Population	# of individuals
ASW	African ancestry in Southwest USA	49
CEU	Utah residents with Northern and Western European ancestry from the CEPH collection	112
CHB	Han Chinese in Beijing, China	84
CHD	Chinese in Metropolitan Denver, Colorado	85
GIH	Gujarati Indians in Houston, Texas	88
JPT	Japanese in Tokyo, Japan	86
LWK	Luhya in Webuye, Kenya	90
MEX	Mexican ancestry in Los Angeles, California	50
MKK	Maasai in Kinyawa, Kenya	143
TSI	Toscani in Italia	88
YRI	Yoruba in Ibadan, Nigeria	113

Supplementary Table 3| Assessment of GWAS findings from SPARK dataset related to Table 1.

see Excel file;
SNPs with P-value < 1×10^{-4} in SPARK GWAS are shown.

Supplementary Table 4| ASD risk loci have pleiotropic effects on various phenotypes

see Excel file;

Supplementary Table 5| Association results across populations

			EUR			AFR			EAS		
SNP	EA	OA	EAF	OR (95%CI)	P	EAF	OR (95%CI)	P	EAF	OR (95%CI)	P
rs716219	T	C	0.34	1.10 (1.03 - 1.17)	0.004	0.19	2.51 (0.87 - 7.29)	0.090	0.36	1.26 (0.79 - 2.01)	0.323
rs10099100	C	G	0.33	1.04 (0.98 - 1.11)	0.175	0.39	1.552 (0.81 - 2.98)	0.185	0.02	0.200 (0.02 - 1.66)	0.136
rs60527016	C	T	0.21	0.83 (0.77 - 0.89)	3.64×10^{-07}	0.03	0.32 (0.03 - 3.21)	0.330	0.29	1.00 (0.60 - 1.65)	0.993
rs112436750	A	AT	0.24	1.10 (1.03 - 1.18)	0.006	0.01	0.0003 (2.58×10^{-22} - 3.59×10^{14})	0.703	<0.01	NA	NA
rs1000177	T	C	0.23	1.06 (0.99 - 1.14)	0.110	0.38	1.46 (0.68 - 3.10)	0.331	0.28	1.31 (0.80 - 2.16)	0.284

Supplementary Table 6| Heritability Enrichment in active enhancers or promoters

see Excel file;

Header information

Header Name	Description
**Category**	Mnemonic
**Epigenome ID**	Epigenome ID (EID)
**Description**	Description of tissue type
**Type**	Tissue type
**Prop._SNPs**	Proportion of SNPs
**Prop._h2**	Proportion of heritability
**Prop._h2_std_error**	Standard error of the proportion of heritability
**Enrichment**	Enrichment (Prop.SNPs heritability)/(Prop. SNPs)
**Enrichment_std_error**	Standard error of the enrichment
**Enrichment_p**	P-value of the enrichment
**FDR**	FDR corrected P-value of the enrichment

Supplementary Table 7| H-MAGMA Gene list (fetal brain)

see Excel file;
Gene position is based on hg19 coordinates.

Header information

Header Name	Description
**GENE**	Ensembl Gene ID
**CHR**	Chromosome
**START**	The annotation boundary of the gene (start)
**STOP**	The annotation boundary of the gene (end)

**NSNPS**	The number of SNPs annotated to that gene
**NPARAM**	The number of relevant parameters used in the model
**N**	The sample size used when analyzing that gene
**ZSTAT**	The Z-value for the gene, based on its P-value
**P**	The gene P-value
**FDR**	FDR corrected gene P-value
**hgnc_symbol**	HGNC symbol
**gene_biotype**	The gene or transcript classification (eg. protein coding)

Supplementary Table 8| Gene ontologies enriched in ASD genes (fetal brain)

see Excel file;

Header information

**Header Name**	**Description**
**query.number**	The number of input query (we tested only one dataset so all should be 1)
**significant**	TRUE if the result is significant at FDR corrected P-value (0.05)
**p.value**	FDR corrected P-value
**term.size**	The number of genes in Gene Ontology (GO) used for hypergeometric test
**query.size**	The number of genes in query used for hypergeometric test
**precision**	Precision
**recall**	Recall
**term.id**	Gene set identifier (GO ID)
**domain**	Sub-ontologies from Gene Ontology<br>MF: Molecular Functions, BP: Biological Process
**subgraph.number**	The number of subset
**term.name**	Gene set name (GO name)

Supplementary Table 9| Gene ontologies enriched in ASD genes (adult brain)

see Excel file;
Header information was described above (Supplementary Table 8)

Supplementary Table 10| H-MAGMA Gene list (adult brain)

see Excel file;
Gene position is based on hg19 coordinates.
Header information was described above (Supplementary Table 7)

Supplementary Table 11| H-MAGMA Gene WGCNA Module

see Excel file;

Header information

Header Name	Description
**Gene ID**	Ensembl Gene ID
**hgnc_symbol**	HGNC symbol
**module**	The name of module

Supplementary Table 12| Genetic correlations between ASD and twelve brain and behavioral phenotypes

Category	Traits	r_g	SE	p	FDR
Psychiatric /Cognitive	Major depressive disorder	0.326	0.031	1.68×10^{-26}	$2.02\times10\text{-}^{25}$
	ADHD	0.319	0.049	2.41×10^{-11}	2.17×10^{-10}
	Intelligence	0.230	0.027	2.08×10^{-17}	2.28×10^{-16}
	Schizophrenia	0.221	0.032	3.18×10^{-12}	3.18×10^{-11}
	Bipolar disorder	0.151	0.042	4.00×10^{-04}	0.002
	Neuroticism	0.140	0.030	2.58×10^{-06}	1.81×10^{-05}
Addiction	Cannabis use	0.292	0.050	5.92×10^{-09}	4.73×10^{-08}
	Cigarettes per day	0.155	0.036	1.76×10^{-05}	1.06×10^{-04}
	Drinks per week	0.033	0.033	0.320	1.00
Degenerative brain disorders	Parkinson's disease	0.021	0.050	0.677	1.00
	Alzheimer's disease	-0.032	0.067	0.632	1.00
Brain size	Intracranial volume	0.004	0.054	0.934	1.00

Supplementary Table 13| MPRA results

see Excel file;

Header information

Header Name	Description
**SNP**	rsID
**Chrom**	Chromosome
**POS**	Position (hg38)
**A1**	A1 allele
**A2**	A2 allele
**Risk**	Risk allele
**OR(A1)**	Odds ratio (A1)
**GWAS P**	GWAS P-value

**Ref**	Reference allele
**Alt**	Alternative allele
**logFC(Alt)**	Estimate of the log2-fold-change corresponding to the Alternative allele
**AveExpr**	Average normalized expression values
**t**	Moderated t-statistic
**P.value**	MPRA raw P-value
**B**	The log odds ratio that the barcode is differentially expressed
**mpraFDR**	FDR corrected MPRA P-value

Supplementary Methods

Genotyping and whole-exome sequencing

Genotyping and sequencing data were generated at the SPARK sites as previously described[8]. Briefly, DNA was extracted from saliva samples and genotyped by Illumina Infinium Global Screening Array-24 v1.0 (GRCh38). For WES, DNA was subjected to target capture using VCRome+PKv2[9] and sequenced using Illumina Novaseq with paired-end 100 bp reads at Regeneron.

SNPs were removed by SPARK prior to download if they were in 56 of 59 ACMG[10] (American College of Medical Genetics and Genomics) recommendations, excluding ASD relevant mutations in *PTEN*, *TSC1*, and *TSC2*. SNPs registered to be pathogenic or likely pathogenic based on ClinVar were also excluded[11].

Pre-imputation quality control for genotype chip data

Prior to sample quality control (QC), we removed SNPs located within Y or mitochondrial chromosomes (Supplementary Fig. 2), or with low genotyping rate (< 0.9). Then, we removed samples with a high missingness (> 0.1) and the discrepancy between self-reported sex and

genotypes. Using WES data, we also checked if there were sample swaps or contaminations (CHIP-MIX or FREE-MIX > 0.8) by VerifyBamID[12] (https://genome.sph.umich.edu/wiki/VerifyBamID), resulting in 10 samples being removed. Additional QC was performed based on the following QC parameters: we retained SNPs with genotyping rate ≥ 0.98, minor allele frequency [MAF] ≥ 0.01, deviation from Hardy-Weinberg equilibrium (HWE) in founders $p \geq 10^{-6}$ for trios, and $p \geq 10^{-6}$ in cases or $p \geq 10^{-10}$ in controls for remaining individuals. For trios, we removed families in which more than 10,000 SNPs have Mendelian-errors. SNPs in which more than four families have Mendelian errors were also excluded.

Genotype phasing and imputation

Phasing was performed using EAGLE v2.4.1[13] (https://data.broadinstitute.org/alkesgroup/Eagle/) within SPARK samples with '--allowRefAltSwap' and '--geneticMapFile=genetic_map_hg38_withX.txt.gz'. The genetic coordinates file (genetic_map_hg38_withX.txt.gz) was distributed by EAGLE. Before making pseudocontrols, we removed two individuals, one each from two pairs of monozygotic twins with Identity-By-Descent (PI_HAT)>0.9, by selecting the individual with lower call rates. Then we defined pseudocontrols by PLINK 1.9[14] (www.cog-genomics.org/plink/1.9/) for trios by selecting the alleles not inherited from the parents to the case[15]. We re-phased all SPARK samples that passed our QC measures with pseudocontrols. Imputation was performed on the Michigan imputation server[16] (https://imputationserver.sph.umich.edu/index.html). Since SPARK participants are genetically diverse, we imputed genotypes using the Trans-Omics for Precision Medicine (TOPMed) Freeze 5b (https://www.nhlbiwgs.org/) reference panel which consists of 125,568 haplotypes from multiple ancestries. Dosage was then converted into a PLINK2 (www.cog-genomics.org/plink/2.0/) pgen retaining phase information. Imputation accuracy relative to WES was assessed using a similar approach to previous work[17] (Supplementary Fig. 4).

Assessment of imputation accuracy

We assessed the accuracy of imputed genotypes by comparing with WES data, using a similar approach to previous work[17] (Supplementary Fig. 4). First, we selected SNPs shared between imputed data and WES for all individuals. We then restricted our analysis to SNPs with 0% missingness in WES. We prepared three sets of SNP lists based on minimac4 imputed R^2. Genotypes were then compared by vcf-compare implemented in VCFtools v0.1.5[18]. The mismatch rate was estimated for each individual as (*the total number of mismatched sites*) / (*the total number of mismatched sites* + *the total number of matched sites*) for sites with MAF ≥ 0.01. Before the final GWAS analysis, we removed poorly imputed individuals with mismatch rate > 3% and selected complete trios (family where the child and both parents survived QC).

Meta-analysis with iPSYCH-PGC study

GWAS summary statistics from Grove *et al*, (iPSYCH-PGC study[1]) were obtained from https://www.med.unc.edu/pgc/shared-methods/data-access-portal/. Chromosomal positions were converted from hg19 to hg38 using R/Bioconductor package liftOver (v.1.4.0)[19] (https://master.bioconductor.org/packages/release/workflows/html/liftOver.html). 15% of SNPs were dropped due to liftOver failure or mismatch with the SPARK dataset (i.e. largely based on small differences in allele frequency, imputation quality, or allelic discrepancy as well the imputation reference panel used). METAL (release 2018-08-28)[20] was used for meta-analysis of our SPARK GWAS (both SPARK full dataset/ European population) and iPSYCH-PGC GWAS summary statistics. The meta-analysis was performed using an inverse-variance weighted fixed-effect design without GC correction.

Investigation of pleiotropic effects for ASD loci

To investigate the pleiotropic effects of identified loci, we tested overlaps with index SNPs from various GWAS for multiple phenotypes available in the NHGRI/EBI GWAS Catalog (downloaded October 22, 2019)[21]. We restricted SNPs to a genome-wide significant level ($P<5.0\times10^{-8}$). Signals

were considered to overlap if the index SNPs of ASD loci and other GWAS loci were in strong linkage disequilibrium (LD) (r^2 > 0.8 in the European ancestry from the 1000 Project [1KG EUR][22]).

Estimating polygenic Risk Score

Polygenic risk scores (PRSs) were calculated based on the iPSYCH-PGC study[1] using PRSice-2[23] (https://www.prsice.info/). To estimate PRS more accurately, we used only SNPs with MAF > 0.01, INFO > 0.9, and that were located outside of MHC regions (chr6:25M-35M). We also used only SNPs tested for association in both iPSYCH and PGC. We identified LD-independent SNPs using clumping with parameters specifying the distance from the index SNP (250 kb) and LD (r^2 >= 0.1) through PRSice-2. The LD was calculated using 1KG EUR phase3. To identify the set of SNPs that best explains risk for ASD, we set multiple P-value thresholds: 5×10^{-8}, 1×10^{-6}, 0.001, 0.01, 0.05, 0.1, 0.2, 0.5 and 1.

For the SPARK dataset, in order to avoid the impact of population stratification on PRS prediction[24–26], we restricted the analysis to European individuals as described above and also selected only one case-pseudocontrol pair from each family. Dosage information was converted to hard-call genotypes using PLINK2 with --geno 0 and --maf 0.05. We estimate the phenotypic variance explained in the SPARK dataset by the iPSYCH-PGC PRS as the Nagelkerke R^2.

For family-type stratified PRS analysis, families were assigned into four groups: 1) multiple children and father/mother are affected, 2) multiple children are affected but mother and father are unaffected, 3) one child and father/mother are affected, 4) only one child is affected. PRS comparisons included 10 genotype PCs as covariates, to control for population stratification, as well as sex.

For sex-stratified PRS, the covariates in the regression included the top 10 PCs of case individuals.

For Parent-of-Origin PRS analysis, the PRS was calculated for paternal and maternal alleles with 10 PCs derived from each parent as covariates. We first generated Oxford-format .haps files containing SNP information (name and position) and individual's phased genotypes by PLINK2 '--haps'. Since this process requires all individuals to have phased genotypes after a hard call, we ran this process for

each trio to obtain the maximum number of SNPs. Then, we classified each SNP in a child as maternal or paternal based on phased information. We note that we excluded SNPs if both parents have the same haplotypes resulting in the unknown parent of origin.

We utilized the same risk alleles described above in other PRS analyses. To reduce the effect of population stratification, we calculated 10 PCs for parents. Paternal PRSs were regressed with 10 PCs from the father, while maternal PRSs were regressed with 10 PCs from the mother.

Heritability Enrichment Analysis

We calculated partitioned heritability enrichment including a baseline model[27] for two datasets as below.

(1) Active enhancer or promoter states present in 127 tissue types[28]. Active enhancer and promoter regions were defined based on chromatin states predicted by chromHMM[29] and included: 'active transcription start site' (state 1), 'flanking active TSS' (state 2), 'genic enhancers' (state 6), and 'enhancers' (state 7) in the core 15-state model [29] (https://egg2.wustl.edu/roadmap/web_portal/chr_state_learning.html).

(2) For differentially accessible regions between CP and GZ, ATAC-seq data were obtained from our previous work[30]. Differential chromatin accessible region was defined if logFC is less than -1 (CP specific) or greater than 1 (GZ specific) at FDR < 0.05. Because the number of differential chromatin accessible regions was different between CP specific peaks (*n* = 17,803) and GZ specific peaks (*n* = 19,260), GZ specific peaks were downsampled to 17,803 by random selection.

We generated annotation files to label SNPs within these annotated regions of the genome. LD scores for each annotation were computed by LDSC based on 1KG EUR phase3. We considered FDR < 0.05 for enrichment P-value as a significance threshold.

Genetic correlation analysis

We tested genetic correlation of ASD with the following phenotypes: ADHD[31], intelligence[32], bipolar disorder[33], schizophrenia[3], major depressive disorder[34], neuroticism[35], cannabis use[36], alcohol (drinks per week)[37], smoking (cigarettes per day)[37], Alzheimer's disease[38], Parkinson's disease[39] and intracranial volume[40]. The .sumstats.gz were prepared in the same way described above. Genetic correlations (r_g) were estimated by LDSC. FDR < 0.05 was considered statistically significant.

H-MAGMA

SNP to Ensembl gene annotation was carried out by Hi-C coupled MAGMA (H-MAGMA) as previously described[41]. In brief, we used Gencode v26 for assigning exonic SNPs and promoter SNPs (2kb upstream to the transcription start sites) to genes based on the location[42]. Intronic and intergenic SNPs were mapped to their target genes based on chromatin interactions to promoters and exons generated by fetal brain and adult brain Hi-C[2,43]. Using this gene-SNP relationship as input, we ran MAGMA (v1.0.7)[44] to aggregate SNP-based P-values to gene-based P-values. We set FDR < 0.1 as the significance threshold.

H-MAGMA gene list was used for further functional analyses including gene ontology enrichment analysis and investigation of developmental trajectory profiles. We primarily focused on H-MAGMA results using fetal brain Hi-C given partitioned heritability enrichment in regulatory elements present during this time period. For expression analysis including expression trajectory (described below), we combined fetal and adult brain H-MAGMA results to ensure that the prenatal enrichment is not driven by using only fetal brain Hi-C data[2].

Gene ontology enrichment analysis for H-MAGMA ASD genes

Gene ontology enrichment analysis was performed using g:Profiler (v0.6.7)[45] (https://biit.cs.ut.ee/gprofiler/) with the "ordered list" option in which all genes were ranked based on P-value from H-MAGMA gene-based test. We selected 18,494 protein-coding genes that were detected in the H-MAGMA gene list and not located within the MHC region (chr6:25M-35M) as the background. We tested enrichment within the Gene Ontology Molecular Functions (MF) and Biological

Process (BP) categories. Gene ontology terms that had less than 5 genes overlapped with query were excluded.

Overlap with genes derived from H-MAGMA and other studies

We evaluated convergence between common and rare variant risk factors by overlapping genes derived from H-MAGMA (common variants driven) with genes that harbor rare variation in ASD (rare variant driven; 102 genes identified by the updated version of Transmitted And *De novo* Association [TADA] model[46] that incorporates probability of loss-of-function intolerance score[47,48] for protein-truncating variants and missense badness, PolyPhen-2, constraint score[49] for missense variants, FDR ≤ 0.1)[50]. Since this study was conducted using WES, we only selected protein-coding genes identified by H-MAGMA (263 genes at FDR<0.1, 18,757 protein-coding genes from H-MAGMA annotation) as background. The significance of overlap between common variant implicated genes (H-MAGMA) and rare variant implicated genes[50] was calculated by the hypergeometric test (phyper in R) as below.

$$\text{phyper}(q\text{-}1, m, n, k, \text{lower.tail = FALSE})$$

where q = number of overlapped genes (= 5), m = number of genes hit by rare variants (= 102), n = number of unoverlapped genes (=18,655), k = number of genes identified by H-MAGMA (= 263)

Differentially expressed genes (DEGs) from the ASD post-mortem cortex compared to neurotypical controls was obtained from genome-wide transcriptome data[51]. DEGs were defined if log2FoldChange > 0 (upregulated in individuals with ASD) or log2FoldChange < 0 (downregulated in individuals with ASD) at FDR < 0.05.

Developmental expression profiles of ASD linked genes

Transcriptome data from embryonic brains and adult brain at 15 developmental epochs was obtained from Kang et al.,[52] through dbGap (Accession phs000406.v1.p1). Because we did not find any

enrichment in subcortical tissues by LDSC heritability enrichment, we extracted expression data in the cortex (frontal cortex, temporal cortex, parietal cortex and occipital cortex) from donors with age < 45. The expression level of 397 protein-coding genes identified by adult brains and/or fetal brains based on H-MAGMA (FDR < 0.1) were centered to mean expression level per sample 'scale(center=T,scale=F)' by R. Prenatal and postnatal were defined if age of donor is between 4 post-conceptional week (PCW) and 38 PCW, or after birth to 45 year olds, respectively. Expression levels between the two groups were compared by t-test. More details were described elsewhere[1,41]. To identify gene clusters that show similar expression trajectory across brain development, we performed weighted gene correlation network analysis (WGCNA)[53,54]. Briefly, to calculate network adjacency from raw expression data, soft-thresholding power of 9 was chosen by scale free topology for multiple soft thresholding implemented in pickSoftThreshold function. Then we translated the adjacency into a topological overlap matrix (TOM) and calculated the corresponding dissimilarity. Co-expression modules were identified as clusters using dynamic hierarchical tree-cut algorithms (cutreeDynamic function) with minClusterSize = 30, deepSplit=2.

Construction of a Massively Parallel Reporter Assay (MPRA) Library

Because the top locus (chr8:38.19M - chr8:38.45M) was also detected in the previous schizophrenia GWAS which is better powered, we obtained credible SNPs for the locus based on schizophrenia GWAS results (Supplementary Table S11 in [3]) (Supplementary Fig. 14). Ninety-eight credible SNPs were detected in this locus. We obtained 150bp sequences that flank each credible SNP with the SNP at the center (74bp + 75bp). Because each SNP has risk and protective alleles, this resulted in 196 total alleles to be tested. The restriction sites for MluI (ACGCGT) and KpnI (GGTACC), as well as primer sequences, were placed upstream and downstream of the 150bp sequences. The resulting 200bp library was made on Agilent Microarrays (Agilent Technologies), which was subsequently resuspended in 50uL of Tris-EDTA (TE) buffer (pH 8.0). The library was further diluted to 1/20th of the concentration. We first amplified the diluted library using qPCR (KAPA HiFi HotStart Real-time PCR

Master Mix [Kapa Biosystems]) to find the optimal number of PCR cycles to amplify the library. The resulting multicomponent plot provided the number of cycles (10 cycles) to be used for the amplification (corresponding to 1/4th of the fluorescence to prevent overamplification). After PCR amplification, the resulting library was cleaned up with Zymo Clean and Concentrator Kit (Zymo Research) and diluted to 0.5ng/uL. Twenty base-pair random barcodes (synthesized from IDT) were then added to the libraries using another round of PCR. We first performed qPCR to determine the best number of amplification cycles, and then the random barcodes were added to the libraries via a PCR using NEBNext Q5 Hot Start Hifi (NEB) with 10 cycles. The library was again cleaned up with Zymo Clean and Concentrator Kit and run on a 2% agarose gel to check for the correct size (250bp).

The resulting library with the barcodes was subsequently digested with MluI-HF (NEB), and SpeI-HF (NEB) in 1X Cut-Smart buffer (NEB) for 1 hour at 37℃. The digested library was cleaned up with Zymo Clean and Concentrator Kit. We also digested a previously described MPRA backbone (Doner_eGP2AP_RC)[55] with MluI-HF, and SpeI-HF in 1X Cut-Smart buffer with rSAP (NEB). The digested library and backbone were then ligated together at room temperature for 30 minutes using T7 DNA Ligase (NEB) in a 1:3 ratio of Doner_eGP2AP_RC:library. The ligated product was cleaned with Zymo Clean and Concentrator Kit and eluted with molecular biograde water (HyClone). The ligated product (Doner_eGP2AP_RC-library) was transformed into NEB 5-alpha Electrocompetent E. coli (NEB). The E. coli was grown in SOC media for 1 hour at 30℃. The cells were then diluted in units of 10 (undiluted, 1/10, 1/100, 1/1,000, and 1/10,000), and plated on LB plates with 50 µg/mL Kanamycin (VWR). These plates were grown overnight at 30°C. The next day, the number of barcodes per variant was estimated by counting the colonies on 1/10,000 plate. We aimed for >100 barcodes per variant, because we hypothesized that the small effect size of a given variant in gene regulation (based on eQTL) would require many barcodes to reliably estimate the gene regulatory activities. If the estimated barcodes per variant were greater than or equal to 100, then the undiluted ligated plate was scraped and grown in 2L of 50 µg/mL Kanamycin LB miller broth for 8 hours at 30°C in a shaking incubator.

After 8 hours, Doner_eGP2AP_RC-library was isolated using Qiagen plasmid maxi prep kit (Qiagen, Cat# 12163). The isolated Doner_eGP2AP_RC-library was tested for the insert using colony PCR and restriction enzyme digestion (KpnI-HF and PsiI-HF [NEB] for 1 hour at 37°C).

We then mapped the random barcodes to the alleles from Doner_eGP2AP_RC-library. We again used a qPCR to estimate the optimal PCR amplification cycles. Based on the cycles corresponding to 1/4th of the fluorescence on the multicomponent plot, we amplified 4ng of Doner_eGP2AP_RC-library at 10 cycles (KAPA HiFi HotStart Real-time PCR Master Mix (2X)) using P5 and P7 Illumina flow cell adapter primers. The amplified product was cleaned up using Zymo Clean and Concentrator Kit, and the resulting library was sequenced by Illumina Miseq 2x150 at UNC High-throughput Sequencing Facility (HTSF). Barcodes were then mapped to individual alleles via custom scripts (https://github.com/kinsigne/bc_map.git).

We then inserted a minimal promoter (minP) and luciferase (luc2) into Doner_eGP2AP_RC-library, so that the 150bp sequences that contain the variants were located upstream of the minP, and the barcodes are located downstream of luc2. The Doner_eGP2AP_RC-library was digested for 3 hours at 37°C with KpnI-HF and XbaI-HF using 1X Cutsmart Buffer and rSAP. The digested library was gel extracted using QIAquick Gel Extraction Kit (Qiagen). The minP-luc2 insert was generated by digesting pMPRAdonor2 (Addgene plasmid #49353) with KpnI-HF and XbaI-HF using 1X Cutsmart Buffer for 1 hour at 37°C. The Doner_eGP2AP_RC-library and minP-luc2 were then ligated together using T7 DNA ligase in a 1:3 ratio (Doner_eGP2AP_RC-library:minP-luc2). The resulting ligation product (the final MPRA library) was cleaned up with Zymo Clean and Concentrator Kit and subsequently transformed into NEB 5-alpha Electrocompetent E. coli. The E.coli was plated on LB plates, and the barcodes per variant was calculated from the plates as described above. If the estimated barcodes per variant was greater than or equal to 100, then the undiluted ligated plate was scraped and grown as described previously. After 8 hours, the final MPRA library was isolated using

Qiagen plasmid maxi prep kit. We measured the concentration of this library by Qubit 1X DS Broad Range (Invitrogen). We further confirmed the resulting library via restriction digestion (KpnI-HF and PsiI-HF) and PCR.

MPRA

We seeded HEK293 cells (ATCC® CRL-11268™) in 6 wells (total 6 replicates) to be 70-90% confluent at transfection. We used lipofectamine 2000 (Invitrogen cat#11668) with our final MPRA library following manufacture instructions. The media on the plated cells was removed and replaced with Optimem (ThermoFisher Scientific). Per well, we diluted 2.5 ug of the complete MPRA construct and 12uL of lipofectamine 2000 reagent in 150uL of Optimem, respectively. The diluted MPRA construct was then added to the diluted lipofectamine. This mixture was incubated at room temperature for 5 minutes, and then added to each well. RNA was extracted from each well using RNeasy Mini Kit (Qiagen cat#74104) 48 hours after transfection. We performed reverse transcription (SSIV reverse transcriptase [Invitrogen]) using primers that amplify the end of luciferase and random barcodes, which would go on to be used to quantify the barcoded expression. The resulting cDNA was amplified with the first PCR using the number of cycles (10 cycles) determined by qPCR as described above. The amplified cDNA was then cleaned up with Zymo Clean and Concentrator Kit and then run in a 4% E-gel (Invitrogen) to verify the correct size. This step was followed by a second PCR (10 cycles) that adds on a unique index (6bp) and Illumina sequencing adaptors. The resulting amplified product was cleaned up using Ampure XP beads (Beckman Coulter) to clean out primers and primer dimers.

As MPRA measures RNA to DNA ratio, we also generated DNA libraries for the final MPRA library used for the transfection of HEK293 cells. The barcode region of the plasmid was amplified via PCR using 100ng of plasmid DNA with 8 cycles. The resulting PCR product was gel extracted from a 1.8% agarose gel using QIAquick Gel Extraction Kit. A second PCR was followed to add on a unique index (6bp) and Illumina sequencing adaptors with 6 PCR cycles. The resulting amplified product was

cleaned up using Ampure XP beads. This final MPRA DNA-seq and RNA-seq libraries were sequenced by Illumina Hiseq 2500 2x150 at UNC HTSF.

MPRA analysis

Because 20bp barcodes may have a larger impact on gene regulation than an allelic difference, we first aggregated RNA barcode counts by taking the sum of RNA barcode counts for a given allele to obtain summarized allelic expression. To control for transfection efficiency and barcode dispersion during cloning, we also aggregated DNA barcode counts. The resulting RNA barcode counts for protective and risk alleles in a total of six replicates were compared against the corresponding plasmid DNA barcodes using an mpra package in R[56,57] (https://github.com/hansenlab/mpra), which yielded allelic expression differences for 98 variants tested. To find the causal regulatory variant out of 98 candidate variants, a simple selection was performed based on the false discovery rate (FDR) and log fold change (logFC) values that represent significance of allelic expression differences. We used FDR<0.01 and |logFC|>1.5 (which represents >50% of up/downregulation mediated by an allelic difference) as a selection criterion, which resulted in two variants: rs7001340 and rs16887340. Among them, rs7001340 was the top variant by P-value. Related figures are provided in Supplementary Fig. 16.

Functional annotation of rs7001340 locus with multi-omic datasets

To investigate the target genes affected by allelic variation at rs7001340, we used two expression quantitative loci (eQTL) data set derived from fetal brain tissues[58] and adult brain tissues[2]. Bulk fetal cortical wall eQTL data described in a previous publication[58], was re-analyzed in this study with the following modifications: (1) here we used a linear mixed model implemented in EMMAX to more stringently control for population stratification, and (2) here we add 23 more donors to the analysis because these donors were genotyped after the publication of the previous manuscript. rRNA-depleted RNA-seq data from flash frozen human fetal brain cortical wall tissues derived from

235 donors at 14-21 PCW were used for eQTL analysis. We used only genes which are expressed in more than 5% of donors with at least 10 counts in the analysis. Genomic DNA was extracted from human fetal brain cortical wall tissues derived from 235 donors at 14-21 PCW. Each donor tissue was genotyped on a dense array (Illumina Omni 2.5+Exome) and imputed to a common reference panel (1KG). Variants were retained in the analysis if they satisfied the following conditions: there were at least 2 heterozygous donors and no homozygous minor allele donors, or there were at least 2 minor allele homozygous donors. We performed cis-eQTL analysis in order to test ssociation between each gene's VST normalized expression value and variants within ±1 Mb window of transcription start site of each gene by implementing linear mixed model association software, EMMAX[59]. Candidate marker excluded IBS kinship matrix was generated with emmax-kin function (-v -h -s -d 10), and used as a random effect in the linear mixed model for association test. In addition to kinship matrix, 10 MDS components of genotype, sex, and first 10 PCs of gene expression were included in the covariate matrix as fixed effects. Nominal P-values from each association were corrected for multiple testing using the Benjamini Hochberg FDR correction, and associations with lower than 5% FDR threshold value were considered as significant.

For SPARK, we calculated LD based on parents of cases. Since donors of fetal brain tissues were from multiple ancestries[58], LD between rs7001340 and SNPs in the locus were calculated based on those samples. Adult brain eQTL visualization used LD from 1KG EUR for eQTL in the adult brain because these donors were largely European.

To map chromatin accessible regions for cultured human neural progenitor cells and their differentiated neuronal progeny, we used the assay for transposase-accessible chromatin sequencing data (73 donors in progenitors, 61 donors in neurons) (ATAC-seq)[60]. Peaks were called by MACS2[61] (https://github.com/taoliu/MACS) with --nolambda --nomodel parameters and removed if the region was overlapped with ENCODE blacklisted regions (http://hgdownload.cse.ucsc.edu/goldenPath/hg19/encodeDCC/wgEncodeMapability/wgEncodeDacMapabilityConsensusExcludable.bed.gz)[42]. To obtain high confidence peaks (40% of samples support

the peak) we ran R/Bioconductor package DiffBind v2.4.8[62,63] (https://bioconductor.org/packages/release/bioc/html/DiffBind.html) as described in the previous report[30]. To find differentially accessible peaks across cell type we controlled for donor differences, the statistical model included a regressor for cell type (progenitor or neuron) and a factor regressor of donor ID.

Open chromatin region in HEK293T cells was obtained from publicly available data (GSM1008573)[4] (https://www.ncbi.nlm.nih.gov/geo/query/acc.cgi?acc=GSM1008573). Data processing of this data was described elsewhere (https://www.ncbi.nlm.nih.gov/geo/query/acc.cgi?acc=GSE32970).

Transcription factor binding (TFB) motifs disrupted by rs7001340 were predicted using R/Bioconductor package motifbreakR (v1.14.0)[5] (https://bioconductor.org/packages/release/bioc/html/motifbreakR.html) for TFB motifs retrieved by MotifDb (v1.26.0)[64] (http://bioconductor.org/packages/release/bioc/html/MotifDb.html). For each TFB nucleotide, disruption scores were calculated based on comparison with position probability metrics from the reference genome 'BSgenome.Hsapience.UCSC.hg38'. Sequence logos were plotted using R/Bioconductor package seqLogo (v1.50.0)[6] (https://bioconductor.org/packages/release/bioc/html/seqLogo.html).

References

1. Grove, J. *et al.* Identification of common genetic risk variants for autism spectrum disorder. *Nat. Genet.* **51**, 431–444 (2019).
2. Wang, D. *et al.* Comprehensive functional genomic resource and integrative model for the human brain. *Science* **362**, (2018).
3. Pardiñas, A. F. *et al.* Common schizophrenia alleles are enriched in mutation-intolerant genes and in regions under strong background selection. *Nat. Genet.* **50**, 381–389 (2018).
4. ENCODE Project Consortium. An integrated encyclopedia of DNA elements in the human genome. *Nature* **489**, 57–74 (2012).

5. Coetzee, S. G., Coetzee, G. A. & Hazelett, D. J. motifbreakR: an R/Bioconductor package for predicting variant effects at transcription factor binding sites. *Bioinformatics* **31**, 3847–3849 (2015).
6. Bembom, O. seqLogo: sequence logos for DNA sequence alignments, R package version 1.50. 0. *Computer software], URL: http://bioconductor. org/biocLite. R* (2019).
7. Polioudakis, D. *et al.* A Single-Cell Transcriptomic Atlas of Human Neocortical Development during Mid-gestation. *Neuron* **103**, 785–801.e8 (2019).
8. Feliciano, P. *et al.* Exome sequencing of 457 autism families recruited online provides evidence for autism risk genes. *NPJ Genom Med* **4**, 19 (2019).
9. Bainbridge, M. N. *et al.* Targeted enrichment beyond the consensus coding DNA sequence exome reveals exons with higher variant densities. *Genome Biol.* **12**, R68 (2011).
10. Kalia, S. S. *et al.* Recommendations for reporting of secondary findings in clinical exome and genome sequencing, 2016 update (ACMG SF v2.0): a policy statement of the American College of Medical Genetics and Genomics. *Genetics in Medicine* vol. 19 249–255 (2017).
11. Landrum, M. J. *et al.* ClinVar: public archive of relationships among sequence variation and human phenotype. *Nucleic Acids Res.* **42**, D980–5 (2014).
12. Jun, G. *et al.* Detecting and estimating contamination of human DNA samples in sequencing and array-based genotype data. *Am. J. Hum. Genet.* **91**, 839–848 (2012).
13. Loh, P.-R. *et al.* Reference-based phasing using the Haplotype Reference Consortium panel. *Nat. Genet.* **48**, 1443–1448 (2016).
14. Chang, C. C. *et al.* Second-generation PLINK: rising to the challenge of larger and richer datasets. *Gigascience* **4**, 7 (2015).
15. Cordell, H. J., Barratt, B. J. & Clayton, D. G. Case/pseudocontrol analysis in genetic association studies: A unified framework for detection of genotype and haplotype associations, gene-gene and gene-environment interactions, and parent-of-origin effects. *Genet. Epidemiol.* **26**, 167–185 (2004).

16. Das, S. *et al.* Next-generation genotype imputation service and methods. *Nat. Genet.* **48**, 1284–1287 (2016).

17. Sariya, S. *et al.* Rare Variants Imputation in Admixed Populations: Comparison Across Reference Panels and Bioinformatics Tools. *Front. Genet.* **10**, 239 (2019).

18. Danecek, P. *et al.* The variant call format and VCFtools. *Bioinformatics* **27**, 2156–2158 (2011).

19. Bioconductor Package Maintainer. *liftOver: Changing genomic coordinate systems with rtracklayer::liftOver*. (2018).

20. Willer, C. J., Li, Y. & Abecasis, G. R. METAL: fast and efficient meta-analysis of genomewide association scans. *Bioinformatics* **26**, 2190–2191 (2010).

21. Buniello, A. *et al.* The NHGRI-EBI GWAS Catalog of published genome-wide association studies, targeted arrays and summary statistics 2019. *Nucleic Acids Res.* **47**, D1005–D1012 (2019).

22. Abecasis, G. R. *et al.* An integrated map of genetic variation from 1,092 human genomes. *Nature* **491**, 56–65 (2012).

23. Choi, S. W. & O'Reilly, P. F. PRSice-2: Polygenic Risk Score software for biobank-scale data. *Gigascience* **8**, (2019).

24. Martin, A. R. *et al.* Human Demographic History Impacts Genetic Risk Prediction across Diverse Populations. *Am. J. Hum. Genet.* **100**, 635–649 (2017).

25. Reisberg, S., Iljasenko, T., Läll, K., Fischer, K. & Vilo, J. Comparing distributions of polygenic risk scores of type 2 diabetes and coronary heart disease within different populations. *PLoS One* **12**, e0179238 (2017).

26. Martin, A. R. *et al.* Clinical use of current polygenic risk scores may exacerbate health disparities. *Nat. Genet.* **51**, 584–591 (2019).

27. Finucane, H. K. *et al.* Partitioning heritability by functional annotation using genome-wide association summary statistics. *Nat. Genet.* **47**, 1228–1235 (2015).

28. Roadmap Epigenomics Consortium *et al.* Integrative analysis of 111 reference human epigenomes. *Nature* **518**, 317–330 (2015).

29. Ernst, J. *et al.* Mapping and analysis of chromatin state dynamics in nine human cell types. *Nature* **473**, 43–49 (2011).

30. de la Torre-Ubieta, L. *et al.* The Dynamic Landscape of Open Chromatin during Human Cortical Neurogenesis. *Cell* **172**, 289–304.e18 (2018).

31. Demontis, D. *et al.* Discovery of the first genome-wide significant risk loci for attention deficit/hyperactivity disorder. *Nat. Genet.* **51**, 63–75 (2019).

32. Savage, J. E. *et al.* Genome-wide association meta-analysis in 269,867 individuals identifies new genetic and functional links to intelligence. *Nat. Genet.* **50**, 912–919 (2018).

33. Stahl, E. A. *et al.* Genome-wide association study identifies 30 loci associated with bipolar disorder. *Nat. Genet.* **51**, 793–803 (2019).

34. Howard, D. M. *et al.* Genome-wide meta-analysis of depression identifies 102 independent variants and highlights the importance of the prefrontal brain regions. *Nat. Neurosci.* **22**, 343–352 (2019).

35. Nagel, M. *et al.* Meta-analysis of genome-wide association studies for neuroticism in 449,484 individuals identifies novel genetic loci and pathways. *Nat. Genet.* **50**, 920–927 (2018).

36. Pasman, J. A. *et al.* GWAS of lifetime cannabis use reveals new risk loci, genetic overlap with psychiatric traits, and a causal influence of schizophrenia. *Nat. Neurosci.* **21**, 1161–1170 (2018).

37. Liu, M. *et al.* Association studies of up to 1.2 million individuals yield new insights into the genetic etiology of tobacco and alcohol use. *Nat. Genet.* **51**, 237–244 (2019).

38. Jansen, I. E. *et al.* Genome-wide meta-analysis identifies new loci and functional pathways influencing Alzheimer’s disease risk. *Nat. Genet.* **51**, 404–413 (2019).

39. Nalls, M. A. *et al.* Expanding Parkinson’s disease genetics: novel risk loci, genomic context, causal insights and heritable risk. *Genetics* 223 (2018).

40. Adams, H. H. H. *et al.* Novel genetic loci underlying human intracranial volume identified through genome-wide association. *Nat. Neurosci.* **19**, 1569–1582 (2016).

41. Sey, N. Y. A. *et al.* A computational tool (H-MAGMA) for improved prediction of brain-disorder risk

genes by incorporating brain chromatin interaction profiles. *Nat. Neurosci.* (2020) doi:10.1038/s41593-020-0603-0.

42. Harrow, J. *et al.* GENCODE: the reference human genome annotation for The ENCODE Project. *Genome Res.* **22**, 1760–1774 (2012).
43. Won, H. *et al.* Chromosome conformation elucidates regulatory relationships in developing human brain. *Nature* **538**, 523–527 (2016).
44. de Leeuw, C. A., Mooij, J. M., Heskes, T. & Posthuma, D. MAGMA: generalized gene-set analysis of GWAS data. *PLoS Comput. Biol.* **11**, e1004219 (2015).
45. Reimand, J., Kull, M., Peterson, H., Hansen, J. & Vilo, J. g:Profiler—a web-based toolset for functional profiling of gene lists from large-scale experiments. *Nucleic Acids Res.* **35**, W193–W200 (2007).
46. He, X. *et al.* Integrated model of de novo and inherited genetic variants yields greater power to identify risk genes. *PLoS Genet.* **9**, e1003671 (2013).
47. Lek, M. *et al.* Analysis of protein-coding genetic variation in 60,706 humans. *Nature* **536**, 285–291 (2016).
48. Kosmicki, J. A. *et al.* Refining the role of de novo protein-truncating variants in neurodevelopmental disorders by using population reference samples. *Nat. Genet.* **49**, 504–510 (2017).
49. Samocha, K. E. *et al.* Regional missense constraint improves variant deleteriousness prediction. *bioRxiv* 148353 (2017) doi:10.1101/148353.
50. Kyle Satterstrom, F. *et al.* Large-scale exome sequencing study implicates both developmental and functional changes in the neurobiology of autism. *bioRxiv* 484113 (2019) doi:10.1101/484113.
51. Parikshak, N. N. *et al.* Genome-wide changes in lncRNA, splicing, and regional gene expression patterns in autism. *Nature* **540**, 423–427 (2016).
52. Kang, H. J. *et al.* Spatio-temporal transcriptome of the human brain. *Nature* **478**, 483–489 (2011).

53. Langfelder, P. & Horvath, S. WGCNA: an R package for weighted correlation network analysis. *BMC Bioinformatics* **9**, 559 (2008).

54. Werling, D., Pochareddy, S., Choi, J., An, J. Y. & Sheppard, B. Whole-genome and RNA sequencing reveal variation and transcriptomic coordination in the developing human prefrontal cortex. *bioRxiv* (2019).

55. Davis, J. E., Insigne, K. D., Jones, E. M., Hastings, Q. B. & Kosuri, S. Multiplexed dissection of a model human transcription factor binding site architecture. *bioRxiv* 625434 (2019) doi:10.1101/625434.

56. Law, C. W., Chen, Y., Shi, W. & Smyth, G. K. voom: Precision weights unlock linear model analysis tools for RNA-seq read counts. *Genome Biol.* **15**, R29 (2014).

57. Myint, L., Avramopoulos, D. G., Goff, L. A. & Hansen, K. D. Linear models enable powerful differential activity analysis in massively parallel reporter assays. *BMC Genomics* **20**, 209 (2019).

58. Walker, R. L. *et al.* Genetic Control of Expression and Splicing in Developing Human Brain Informs Disease Mechanisms. *Cell* **179**, 750–771.e22 (2019).

59. Kang, H. M. *et al.* Efficient control of population structure in model organism association mapping. *Genetics* **178**, 1709–1723 (2008).

60. Liang, D. *et al.* Cell-type specific effects of genetic variation on chromatin accessibility during human neuronal differentiation. *bioRxiv* 2020.01.13.904862 (2020) doi:10.1101/2020.01.13.904862.

61. Zhang, Y. *et al.* Model-based analysis of ChIP-Seq (MACS). *Genome Biol.* **9**, R137 (2008).

62. Stark, R. & Brown, G. DiffBind: differential binding analysis of ChIP-Seq peak data. *R package version* **100**, 4–3 (2011).

63. Ross-Innes, C. S. *et al.* Differential oestrogen receptor binding is associated with clinical outcome in breast cancer. *Nature* **481**, 389–393 (2012).

64. Shannon, P. & Richards, M. MotifDb: An annotated collection of protein-DNA binding sequence motifs. R package version 1.26. 0. *Computer software], URL: http://bioconductor. org/biocLite. R*

(2019).
